# Morphological and ecological divergence of *Lilium* and *Nomocharis* within the Hengduan Mountains and Qinghai-Tibetan Plateau may result from habitat specialization and hybridization

**DOI:** 10.1186/s12862-015-0405-2

**Published:** 2015-07-29

**Authors:** Yun-Dong Gao, AJ Harris, Xing-Jin He

**Affiliations:** Key Laboratory of Bio-Resources and Eco-Environment of Ministry of Education, College of Life Science, Sichuan University, Chengdu, China; Key Laboratory of Mountain Ecological Restoration and Bioresource Utilization & Ecological Restoration Biodiversity Conservation Key Laboratory of Sichuan Province, Chengdu Institute of Biology, Chinese Academy of Sciences, Chengdu, 610041 China; Department of Botany, Oklahoma State University, 301 Physical Sciences, Stillwater, OK 74078-3013 USA

**Keywords:** Ancestral state reconstruction, biogeography, Divergence time, *Lilium*, *Nomocharis*, Hengduan Mountains, Qinghai-Tibetan Plateau

## Abstract

**Background:**

Several previous studies have shown that some morphologically distinctive, small genera of vascular plants that are endemic to the Qinghai-Tibetan Plateau and adjacent Hengduan Mountains appear to have unexpected and complex phylogenetic relationships with their putative sisters, which are typically more widespread and more species rich. In particular, the endemic genera may form one or more poorly resolved paraphyletic clades within the sister group despite distinctive morphology. Plausible explanations for this evolutionary and biogeographic pattern include extreme habitat specialization and hybridization. One genus consistent with this pattern is *Nomocharis* Franchet. *Nomocharis* comprises 7–15 species bearing showy-flowers that are endemic to the H-D Mountains. *Nomocharis* has long been treated as sister to *Lilium* L., which is comprised of more than 120 species distributed throughout the temperate Northern Hemisphere. Although *Nomocharis* appears morphologically distinctive, recent molecular studies have shown that it is nested within *Lilium*, from which is exhibits very little sequence divergence. In this study, we have used a dated molecular phylogenetic framework to gain insight into the timing of morphological and ecological divergence in *Lilium*-*Nomocharis* and to preliminarily explore possible hybridization events. We accomplished our objectives using dated phylogenies reconstructed from nuclear internal transcribed spacers (ITS) and six chloroplast markers.

**Results:**

Our phylogenetic reconstruction revealed several *Lilium* species nested within a clade of *Nomocharis*, which evolved ca. 12 million years ago and is itself nested within the rest of *Lilium*. Flat/open and horizon oriented flowers are ancestral in *Nomocharis*. Species of *Lilium* nested within *Nomocharis* diverged from *Nomocharis* ca. 6.5 million years ago. These *Lilium* evolved recurved and campanifolium flowers as well as the nodding habit by at least 3.5 million years ago. *Nomocharis* and the nested *Lilium* species had relatively low elevation ancestors (<1000 m) and underwent diversification into new, higher elevational habitats 3.5 and 5.5 million years ago, respectively. Our phylogeny reveals signatures of hybridization including incongruence between the plastid and nuclear gene trees, geographic clustering of the maternal (i.e., plastid) lineages, and divergence ages of the nuclear gene trees consistent with speciation and secondary contact, respectively.

**Conclusions:**

The timing of speciation and ecological and morphological evolutionary events in *Nomocharis* are temporally consistent with uplift in the Qinghai-Tibetan Plateau and of the Hengduan Mountains 7 and 3–4 million years ago, respectively. Thus, we speculate that the mountain building may have provided new habitats that led to specialization of morphological and ecological features in *Nomocharis* and the nested *Lilium* along ecological gradients. Additionally, we suspect that the mountain building may have led to secondary contact events that enabled hybridization in *Lilium-Nomocharis*. Both the habitat specialization and hybridization have probably played a role in generating the striking morphological differences between *Lilium* and *Nomocharis*.

**Electronic supplementary material:**

The online version of this article (doi:10.1186/s12862-015-0405-2) contains supplementary material, which is available to authorized users.

## Background

The Hengduan Mountains (H-D Mountains) are located in southwestern China east of the Qinghai-Tibetan Plateau (QTP) and represent one of the world’s most biodiverse regions [1]. Many endemic vascular plant species of the H-D Mountains exhibit high levels of morphological and ecological divergence from their closest, more widespread allies. Thus, the endemics are often treated within their own genera. However, molecular phylogenetic studies have revealed that the some of these endemic genera are nested within the widespread ones. Examples include representatives of Asteraceae (*Sinacalia*), Brassicaceae (*Solms-laubachia*), Liliaceae (*Lloydia*), Primulaceae (*Pomatosace*), Genetianaceae (*Lomatogoniopsis*), and Amaryllidaceae (*Milula*) (see more detail information in Table 1, [2–8]). The contrasting morphological diversity and nested phylogenetic status of genera in the H-D Mountains may result from extreme habitat specialization and/or hybridization events. The H-D mountains provide many unique habitats due to their topographic complexity [9], while repeated phases of uplift of the mountain range may have enabled opportunities for hybridization [10, 11] via secondary contact. Continued research is needed to better understand the mechanisms driving morphological diversity of vascular plants within the H-D Mountains.

**Table 1:** Morphologically distinctive plant species that are endemic to the QTP but phylogenetically indistinct (i.e., nested within) from allies

Endemic OTU(s)	Phylogenetically indistinct allies	Distinctive morphology of endemic	Morphology of allies	Geographic range of allies	Family	Reference
*Lomatogoniopsis* T. N. Ho & S. W. Liu	*Lomatogonium* A. Braun	2n = 12; Petals bearing one nectary each; Nectaries appendaged, not in pits	2n = 18; Petals bearing two nectarines each; Nectaries not appendaged, in pits	Throughout the temperate Northern Hemisphere	Gentianaceae	[9, 84]
*Milula* Prain	*Allium* L.	*n* = 10; inflorescence spicate; sepals fused over 1/3 or more of length	*n* = 16 or multiples; inflorescence umbellate; sepals free or fused only at base	Throughout the Northern Hemisphere and in Africa, and Central and South America	Liliaceae	[9, 85]
*Parapteropyrum*	*Fagopyrum*	woody; flowers bisexual	herbaceous; flowers monoecious	QTP and adjacent regions to the south and east	Polygonaceae	[86]
*Parasenecio* W. W. Smith & J. Small	*Sinacalia* H. Robinson & Brettell	capitula discoid; roots not tuberous	capitula radiate; roots tuberous	Throughout temperate China	Asteraceae	[9, 87]
*Pomatosace* Maximowicz	*Androsace* L.	fruit capsule operculate	fruit capsule opening along longitudinal slits	Temperate Northern Hemisphere except eastern North America and temperate South America	Primulaceae	[9, 88, 89]
*Solms-laubachia* Muschler	*Parrya* R. Brown; *Desideria* Pampanini	unique suite of characters	unique suite of characters	Temperate, subarctic, and arctic areas in eastern and central Asia and North America	Brassicaceae	[3, 9, 90, 91]

**Fig. 1:**
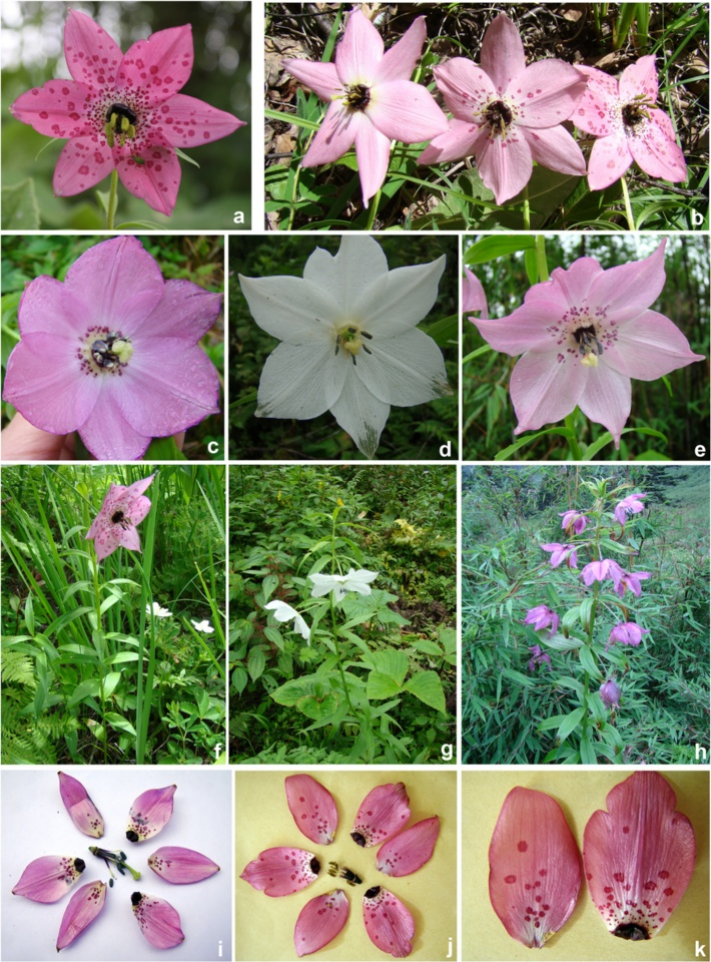
Pictures of *Nomocharis aperta* in western Yunnan: (**a**-**c**), population from Zhongdian, Yunnan showed spot variation; (**c**-**e**), population of Fugong, Yunnan showed variations in tepal color; (**f**-**h**), habits of *N. aperta* under different habitats; (**i**-**j**), anatomical pictures showed two types of *N. aperta* from Zhongdian and Fugong, as well as a comparison of outer and inner tepals

**Fig. 2:**
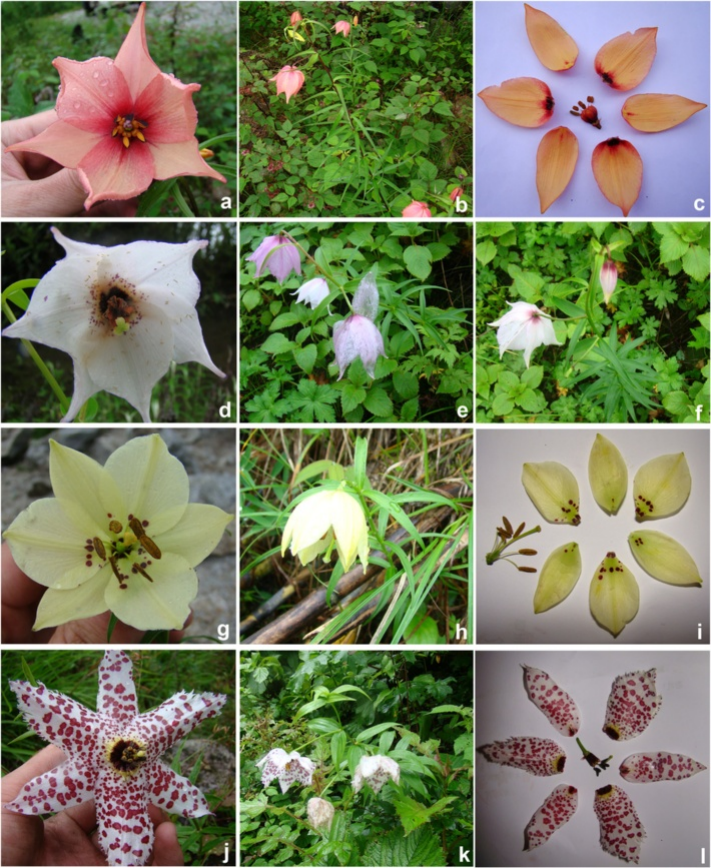
Pictures from western China showing *Nomocharis*: (**a**-**c**), *N. basilissa*; (**d**-**f**), *N. farreri*; (**g**-**i**), *N. gongshanensis*; (**j**-**l**), *N. meleagrina*

The *Lilium*-*Nomocharis* complex represents an exceptional study system for morphological diversification and hybridization in the H-D Mountains. *Nomocharis* Franchet. is endemic to the H-D Mountains and adjacent QTP. *Nomocharis* appeared somewhat similar to *Lilium* when the former was first described in 1889 [12, 13] but was erected as a new genus because of its highly distinctive open-plate flowers and dark-colored tepal bases with special structures (Figs. 1 and 2) [12–15]. Currently, there are eight recognized species of *Nomocharis*, of which seven are circumscribed in two traditional sections [14, 15], and one is a recently described hybrid species, *N. gongshanensis* Y. D. Gao & X. J. He [16]. Recent molecular phylogenetic studies show strong support for *Nomocharis* nested within *Lilium* [16, 17]. In contrast to *Nomocharis*, *Lilium* comprises approximately 120 species and is widespread throughout the Northern Hemisphere, including areas within the QTP and H-D mountains [18–20].

The goals of our present study are to use a molecular phylogeny as a framework to 1) determine whether the timing of morphological and ecological evolutionary events in *Nomocharis* are consistent with phases of uplift in the H-D Mountains and QTP, and 2) detect additional hybridization events with the *Lilium-Nomocharis* species of the H-D Mountains and QTP.

## Results

### Phylogenetic analyses

**Fig. 3:**
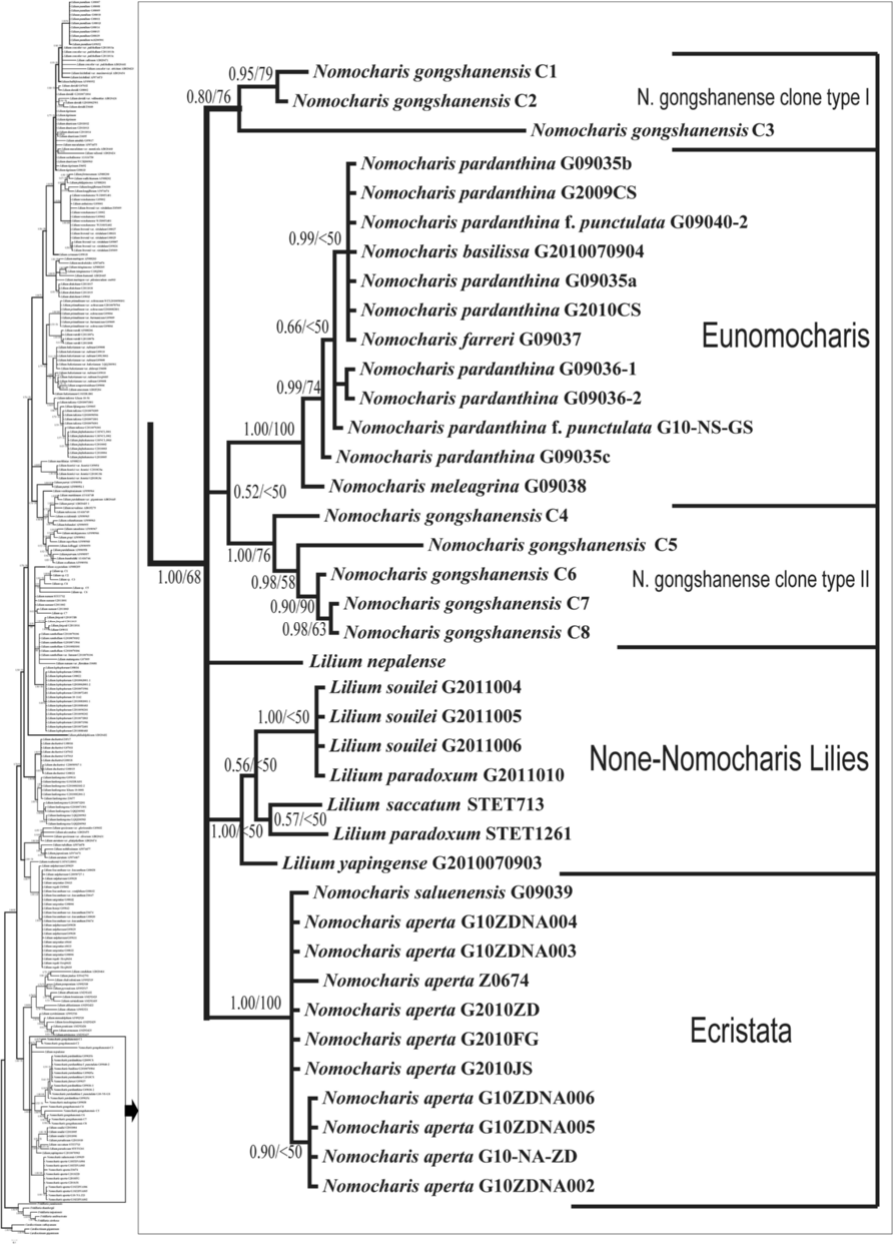
Maximum credibility tree showing monophyletic clade of *Nomocharis* and its relatives reconstructed using Bayesian analysis of ITS data and *Lilium* species from around the world. The position of this clade is indicated on the tree (for details see Additional file 1: Figure S1). Support values shown on braches; Bayesian posterior probabilities (PP) on left and parsimony bootstrap (BS) on right. Clade names based on Balfour [12]

A large ITS dataset confirmed the phylogentic position of *Nomocharis* within *Lilium* and showed no major differences compared with previous studies (e.g., [16, 17, 21]). Our extensive sampling of *Nomocharis* enabled us to resolve three sublclades within the genus: Eunomocharis, Ecristata, and the Non-Nomocharis lilies (*Lilium* species, N-N, hereafter). The Eunomocharis and Ecristata subclades are congruent with traditional classifications based on morphology [13]. The N-N lilies are morphologically divergent from *Nomocharis* and have characteristics more like other *Lilium* (Fig. 3). *Nomocharis* and the N-N lilies are sister to a clade comprised of *Lilium* sect. *Liriotypus* (i.e., European lilies) and that these two clades are sister to the rest of *Lilium* (Additional file 1: Figure S1).

**Fig. 4:**
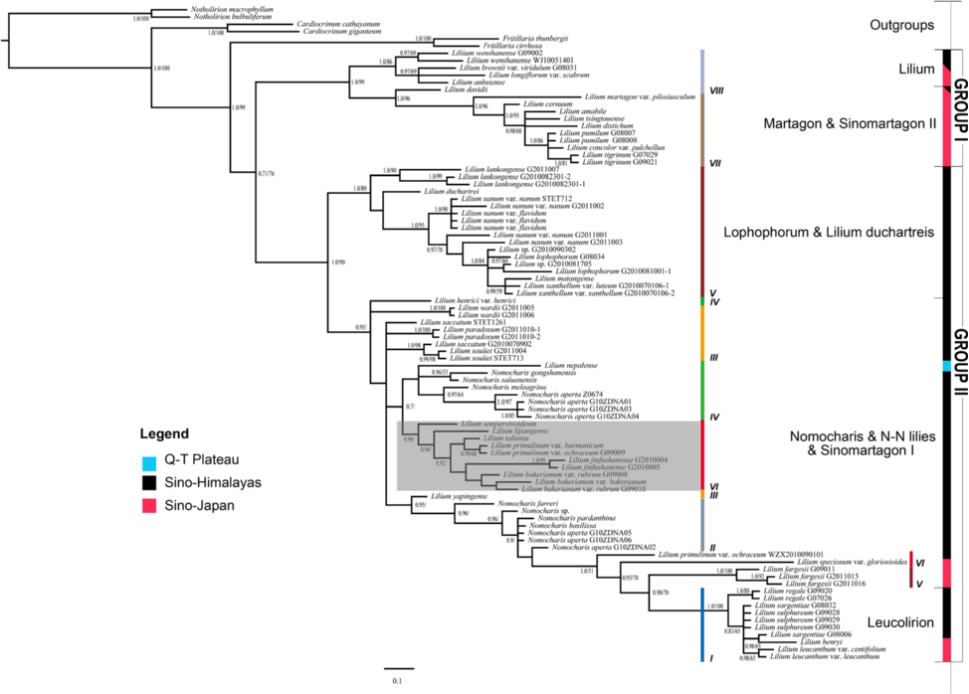
Maximum credibility tree resulting from a Bayesian analysis of combined plastid DNA. Clade names based on Comber [23] and Liang [19]. Distributional areas of clades indicated by color. Support values shown on braches; Bayesian posterior probabilities (PP) on left and parsimony bootstrap (BS) on right. Lineages identified in network (Fig. 5) were also marked for references. The Sinomartagon I clade is highlighted for its conflicting position compared to the ITS result in Additional file 1: Figure S1

Major clades of the plastid consensus trees were the same in the Bayesian and MP reconstructions, so we present only the Bayesian consensus (Fig. 4). The plastid data resolved two large clusters consisting of seven major clades (Fig. 4). Cluster I (PP = 1.00, BS = 99 %) comprised two major clades of species of *Lilium* that are primarily distributed throughout the Sino-Japanese Forest subkingdom [22]. Cluster II (PP = 1.0, BS = 90 %) contained *Nomocharis* and species of *Lilium* that occur within the H-D Mountains and adjacent Himalayas.

Within the plastid phylogeny, *Nomocharis* formed a poorly resolved grade with species of the *Sinomartagon* and *Leucolirion* clades. Most of the species of *Sinomartagon* that associated with *Nomocharis* and the N-N lilies occur in the *Sinomartagon* I clade in the ITS topology and represent all *Sinomartagon* species that inhabit the H-D Mountains and QTP [23, 24]. Despite poor resolution of *Nomocharis* within the plastid phylogeny, the genus roughly comprised its traditionally recognized sections, sects. *Ecristata* and *Eunomocharis*. A clade of *Ecristata* included *N. aperta* accessions and *N. saluenensis*, which have been have been historically treated in the section*.* The *Ecristata* clade also contained clones *N. gongshanensis*, which is the hybrid species, *L. nepalense,* and *N. meleagrina,* which is morphologically similar to species of *Eunomocharis* by having whorled leaves and has traditionally been circumscribed in that section. A grade of sect. *Eunomocharis* also included one accession of *N. aperta* (Franchet) E.H. and *Lilium yapingense*, an N-N lily species.

Overall, *Nomocharis* and the N-N lilies exhibited poorly resolved relationships within cluster II of the plastid phylogeny and did not form a monophyletic group.

### Statistical parsimony network

**Fig. 5:**
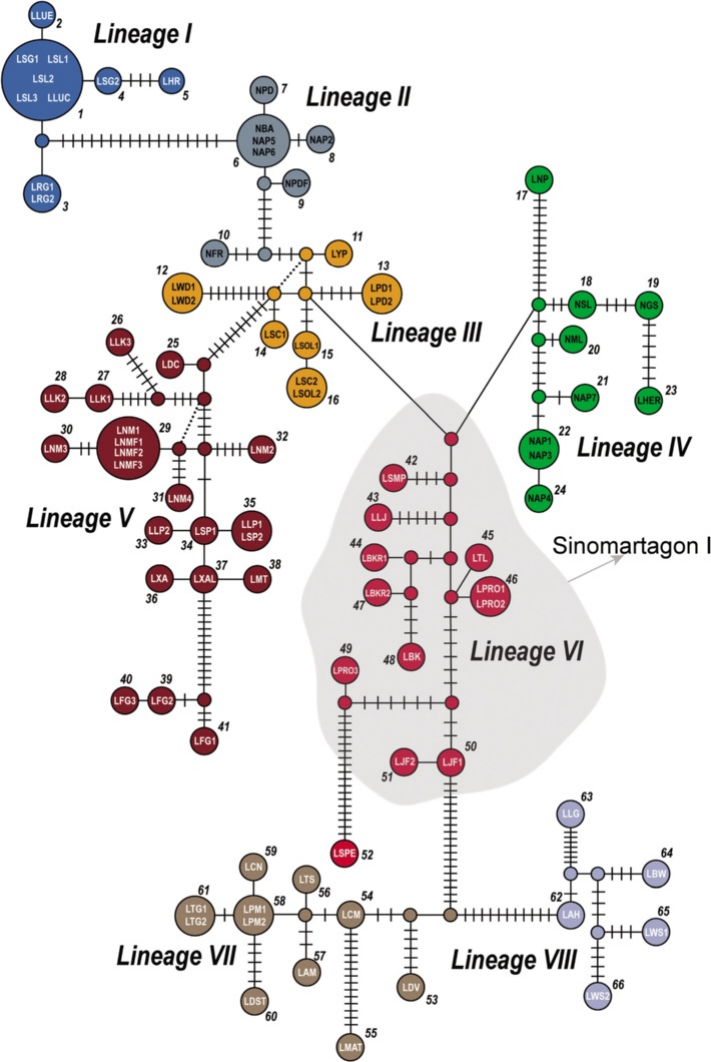
Parsimony network conducted by TCS [58] using combined plastid DNA matrix. Sixty-six haplotypes were identified and clustered in eight lineages with different colors. Circle sizes correspond to the number of taxa possessing the haplotype. Species names are abbreviated by the generic first letter and two or three letters of the species epithet (Table 2). Inferred haplotypes (not present in the data set) are depicted as black lines, and unnamed dots indicated the missing interior haplotypes. The Sinomartagon I clade was highlighted for its conflict position compared to the ITS result in Additional file 1: Figure S1

Our parsimony network was complex but relatively well resolved (Fig. 5). Interior haplotypes and their descendants appear to represent eight lineages, most of which are present in the dichotomously branching plastid phylogeny (Fig. 4). The network supported the plastid tree topology in showing that geographically proximal species have more closely related haplotypes irrespective of morphological similarities or classification in traditional subgenera. Notably, the plastid tree and network also agreed in the placement of *Nomocharis*. In the network, *Nomocharis* was divided into two lineages, II and IV, and separated by Lineage III in which N-N lilies were included (Fig. 5). Haplotypes of the *Nomocharis* and the N-N lilies of lineages III and IV exhibit a shared history with *Sinomartagon* and *Leucolirion* species of lineage VI and VII as well as with species of a Lilium clade (lineage VIII, compare to Fig. 4).

### Divergence time estimate and biogeography inferences

**Fig. 6:**
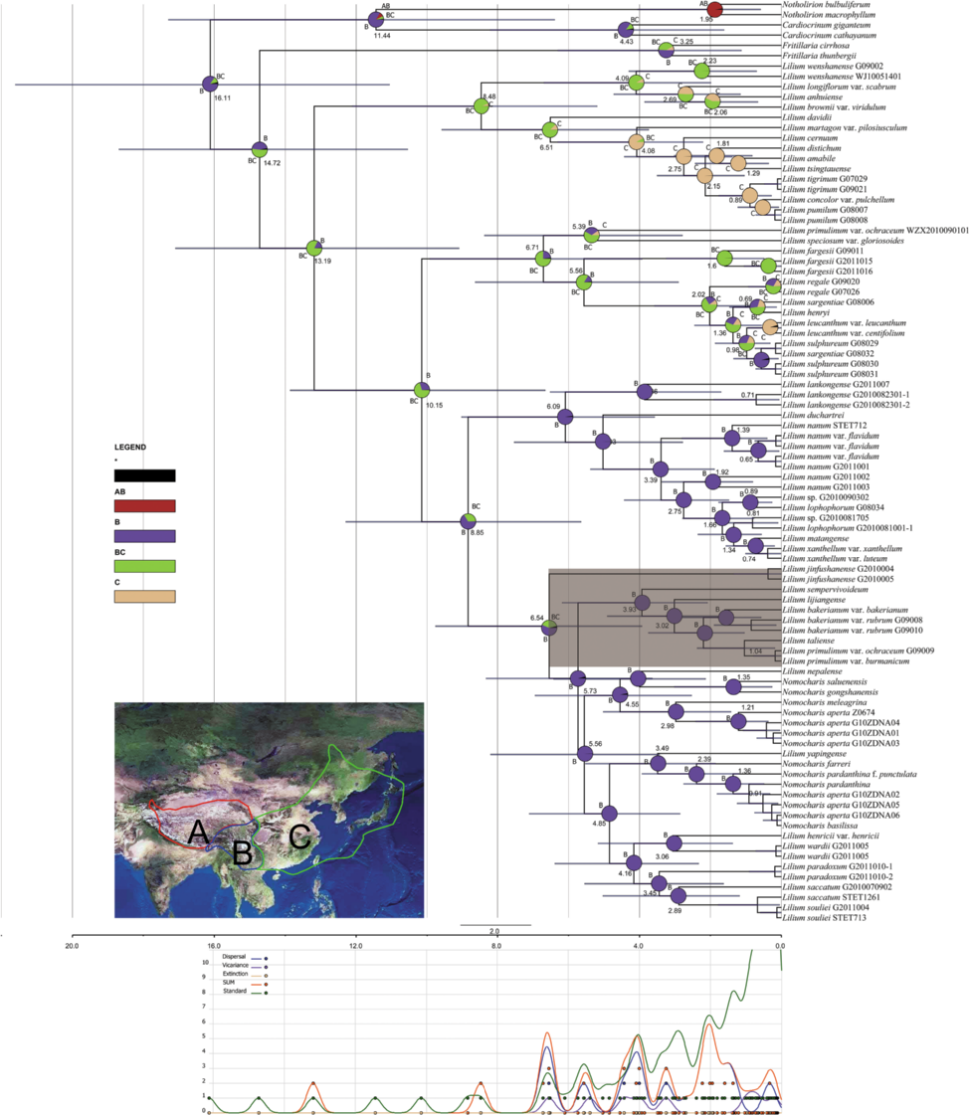
Ultrametric chronograms showing divergence time dating and biogeographic results based on the combined plastid DNA phylogeny. Scale bar at bottom indicating branch length of 2 Mya. Mean divergence age given on nodes. Bars on nodes indicate the 95 % HPD for divergence ages. Pie charts show probabilities of ancestral area reconstructions, colors of pie slices defined in legend. The bottom chart summarized the biogeographic event through time. The Sinomartagon I clade was highlighted for its conflict position compared to the ITS result in Additional file 1: Figure S1

**Fig. 7:**
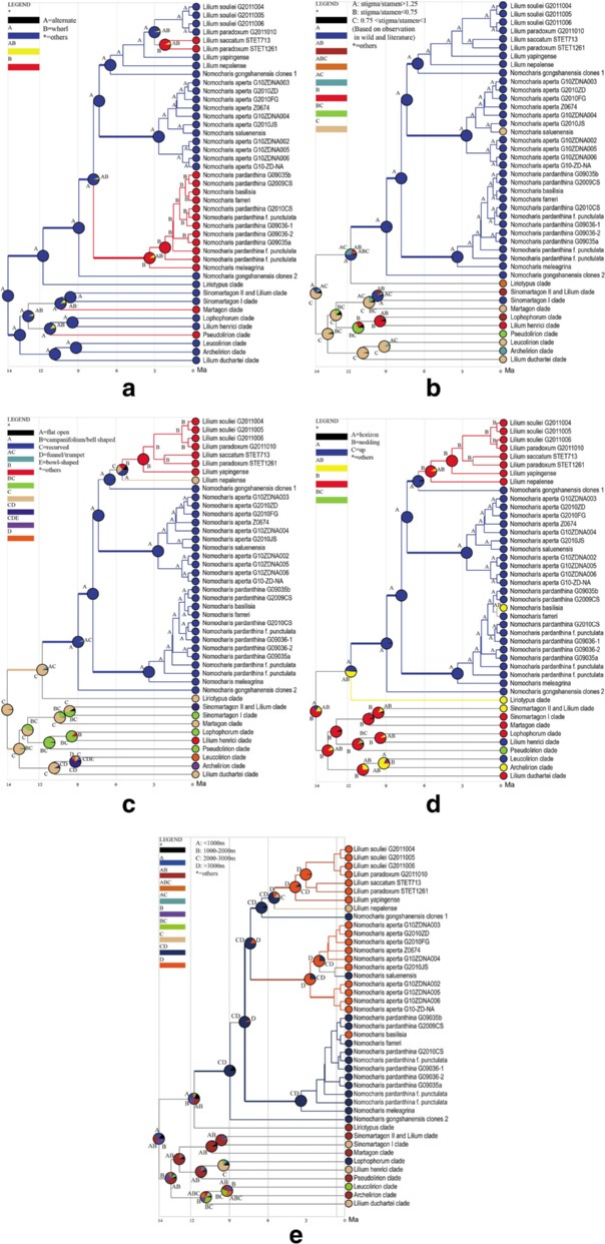
The ancestral state reconstructions of leaf, flower, and ecological characters. Pie charts show probabilities of ancestral area reconstructions, colors of pie slices defined in legend. Reconstructions of **a**, leaf arrangement, **b**, stigma:stamen ratio, **c**, corolla shape, **d**, corolla orientation with respect to the ground, and **e**, elevational range

We performed divergence time dating using two secondary calibration points applied to our ITS plastid dataset. According to dating using the plastid dataset, and we inferred that the last shared ancestor of the *Lilium*-*Nomocharis* occurred around 13.19 Mya and Nomocharis evolved 6.5 Mya (Fig. 6). The ITS dataset recovered a slightly older age of approximately 14 Mya for the last shared ancestor of *Lilium*-*Nomocharis* and ca. 12 Mya for the evolution of *Nomocharis* (Fig. 7). Overall, the ITS dates for major diversification events are older than the plastid dates (Figs. 6 and 7).

The results from Bayesian Binary Method (BBM) of biogeographic analysis show that the last shared ancestor of *Lilium-Nomocharis* arose in the H-D Mountain region (B: 78.4 %; Fig. 6), while the results from the DEC method in Lagrange support a broader ancestral area within the H-D Mountains and the adjacent Sino-Japanese Floristic Subkingdom (SJFS; BC: 21.4 %; Fig. 6). The results obtained from BBM and DEC may not be incongruent because no significant geographic boundary separated the H-D Mountains and the SJFS areas until at least late Miocene (~7 Mya), which is the earliest date postulated for the H-D Mountain uplift [25, 26]. *Lilium*-*Nomocharis* began intensive diversification in the late Miocene (ca. 11–5 Mya, Fig. 6 or ca. 13–6 Mya, Fig. 7). The three *Nomocharis* lineages, *Eunomocharis*, *Ecristata*, and the N-N lilies, originated approximately between ca. 8 Mya (ITS, Fig. 7) and 6 Mya (plastid, Fig. 6) and underwent diversification during the late Pliocene beginning ca. 7–4 Mya (Figs. 6 and 7 respectively).

### Ancestral state reconstruction (ASR)

We performed our ancestral state reconstructions using a reduced ITS dataset and they showed that floral characters were more phylogenetically dependent than vegetative ones (Fig. 7). Leaf arrangement patterns showed the greatest lability within clades (Fig. 7a). Overall whorled leaves arose at least four times in *Lilium*, including two shifts to whorled leaved within *Nomocharis* and the N-N lilies occurring approximately 4 Mya and 2.5 Mya, respectively (Fig. 7). Our results show that nodding flowers with recurved tepals and roughly equal stigma and stamen lengths are most likely the ancestral condition for *Lilium* (Fig. 7b, c, d). Ancestors of *Nomocharis* had longer stigmas than stamen, and this feature also was a synapomorphy within the sympatric Sinomartagon I clade (Fig. 7b). However, one species of *Nomocharis*, *N. saluenensis*, experienced a reversion to the roughly equal condition about 1 Mya (Fig. 7b). There appeared to be a correlation between floral orientation and corolla shape; namely that species with campanifolium and recurved petals have nodding flowers, and species with flat open and funnel/trumpet shaped flowers are horizon in orientation (Fig. 7c, d). This seems to be true among modern species and reconstructed ancestors. Recurved and campanifolium petals and the nodding habit evolved in the last shared ancestor of the N-N lilies around 7.5 Mya, and distinguish them from *Nomocharis*, which retained flat/open flowers and horizon orientation (Fig. 7c, d). The elevation reconstruction indicate that the ancestors of *Nomocharis* and the N-N lilies occurred at low (<1000 m) elevations and that radiations into different elevations habitats occurred around 5.5 Mya in the N-N lilies and around 3.5 Mya in the Ecristata clade of *Nomocharis* (i.e., including *N. aperta* accessions and *N. saluenensis*; Fig. 7e).

## Discussion

### Morphological divergence and habitat specialization

Traditionally, classification of *Lilium* has focused primarily on floral morphology, especially orientation of the flowers with respect to the ground and corolla shape. Thus, nodding flowers and campaniform corollas have been used to support a close relationship between the N-N lilies, which include *L. nepalense*, *L. souliei*, *L. paradoxum*, *L. saccatum* and *L. yapingense* (Additional file 3: Figure S3, Additional file 4: Figure S4), and sect. *Lophophorum* (e.g., *Lilium nanum*, Additional file 4: Figure S4h, k, and *L. lophophorum*, Additional file 3: Figure S3d, e, f, of sect. *Lophophorum*), which shares the same floral features [23]. However, our ITS phylogeny is in contrast to traditional classification of the N-N lilies with sect. *Lophophorum* and shows that the N-N species are nested within *Nomocharis*, which is otherwise monophyletic (Figs. 3, Additional file 1: Figure S1). The N-N lilies share few apparent morphological traits in common with *Nomocharis* and, in particular, lack the unique floral characters that have classically been used to delimit *Nomocharis* from *Lilium*.

N-N lilies and traditional *Nomocharis* may exhibit morphological dissimilarities despite their close evolutionary relationships due to habitat specialization. The N-N lilies may have expanded their habitats into diverse elevations around 5.5 Mya that became available after the last QTP orogeny, which occurred ca. 7 Mya [27, 28] (Fig. 5e). Similarly, uplift of the H-D Mountains probably provided new habitat for an ancestor of the Ecristata clade of *Nomocharis*. Within the QTP, the N-N lilies tend to occupy higher elevations than the Nomochrais species of the H-D Mountains. Differential adaptations to elevation may explain the strikingly different floral morphology of *Nomocharis* and the N-N species [29]. In particular, the N-N lilies live almost exclusively in alpine meadows. Thus, N-N lilies are exposed to torrential downpours in alpine meadows compared to traditional *Nomocharis* species, which grow in the herbaceous layer beneath bamboo canopies (Additional file 5: Figure S5b, h) [19, 20]. The N-N lilies may have evolved nodding flowers ca. 7.5 Mya during QTP uplift and campaniform corollas as advantageous protections for their delicate reproductive structures against harsh precipitation conditions [30, 31]. Although the nodding, campaniform flowers probably provide protection from rainfall for the N-N lilies, they may also have reduced pollen transfer efficiency as an evolutionary trade-off [13, 14]. In contrast, *Nomocharis* species are probably not limited by the need for protection from heavy rainfall, and may experience higher pollen transfer efficiency by virtue of their horizontally arranged, plate-shaped flowers [13, 14].

The profound effects of habitat specialization within the H-D Mountains and QTP regions on morphology is supported by evidence of convergent evolution among sympatric, distantly related *Lilium-Nomocharis* species. In particular, *Nomocharis* and N-N lilies share some morphological traits in common with species of the *Lophophorum* clade, despite their differences and with which they are sympatric in alpine areas of the QTP. Shared traits especially include inner perianth-segments that have crested or fringed glandular bases (e.g., *L. nanum* and *L. lophophorum* Additional file 6: Figure S6) and that are sometimes anthocyanin rich (e.g., *L. henrici* Additional file 6: Figure S6). These shared morphological traits appear to represent convergent evolution. Morphological convergence within QTP alpine plant genera has been noted in other plant genera including in *Androsace* (Primulaceae) [5], *Pseudoeriocoryne* (Asteraceae: Cardueae) [32], *Rheum* (Polygonaceae) [33] and the *Ligularia-Cremanthodium-Parasenecio* complex (Asteraceae) [2]. An alternative explanation for the shared morphology between *Nomocharis* and *Lophophorum* is hybridization. However, the monophyly of *Lophophorum* is supported by both ITS and plastid phylogenies (Figs. 3 and 4). Thus, convergence seems to better explain the morphological similarities and supports habitat specialization of *Nomocharis* and the N-N lilies within the H-D Mountains and QTP.

Detecting the environmental drivers of convergence remain beyond the scope of this study. However, it is noteworthy that many alpine plant groups exhibit floral traits that are well-adapted to the frequent but unpredictable rains experienced in alpine habitats [34–36]. For example, the nodding flower orientation is thought to have evolved to avoid pollen damage and nectar dilution by rainfall [30, 31, 37, 38]. Floral orientation may also be strongly affected by niche features such as the presence and abundance of various types of pollinators. In particular, the horizontal orientation may increase the precision of pollen transfer in bilaterally symmetrical flowers (e.g. *Lilium* and *Nomocharis*) under some pollination syndromes [35, 36, 39]. However, morphological convergence among alpine plants may also be strongly affected by understudied environmental interactions, such as with the intense solar radiation experienced during the daytime in alpine areas or the cold night time temperatures [31]. Overall, morphological convergence within the QTP and H-D Mountains habitats is likely linked to the extreme morphological divergence between QTP and H-D Mountains endemics and their widespread relatives. Thus, morphological convergence among QTP and H-D Mountains species of *Lilium-Nomocharis* and within other plant groups merits more attention in future studies.

### Hybridization

Our ITS and plastid gene trees reveal several signatures of possible hybridization. In particular, the gene trees exhibit incongruence. In the ITS phylogeny, *Nomocharis* and the N-N lilies form a clade in the ITS tree (Fig. 3) that is sister to *Lilium* sect. *Liriotypus*. This is in contrast to the plastid phylogeny, which shows poor resolution of *Nomocharis* and the N-N lilies and places them among species of sects. *Sinomartagon, Martagon* (Fig. 4). Incongruence between nuclear and plastid and nuclear gene trees is known to result from hybridization, but can also result from incomplete lineage sorting, which is common among vascular plants, and horizontal gene transfer, which is not [40, 41].

Another signature of hybridization may be the strong geographic clustering observed in the plastid phylogeny (Fig. 4) among clades, which are distantly related in the nuclear phylogeny (Fig. 3, Additional file 1: Figure S1). The sympatry of clades with closely related plastid genomes is consistent with secondary contact. Moreover, hybridization in *Lilium-Nomocharis* is most likely to occur among species that occur within reasonably close proximity due to the limited dispersability of seeds [42] and typically also of pollen via wind or pollinators [43].

If hybridization did occur between *Nomocharis* (including N-N lilies) and sympatric *Lilium*, it must have occurred following the evolution of the latter, ca. 12 Mya (Fig. 7). If the dates in the plastid phylogeny can be taken to represent the times of contact, then hybridization events occurred in *Nomocharis* 5.73 Mya with *Sinomartagon* and 4.85 Mya with *Leucolirion* species. These events seem to post-date late orogenies of the QTP ca. 7 Mya and pre-date uplift of the H-D Mountains, in the late Neogene (ca. 3.4 Mya, [25, 26]). However, 95 % CIs for the dates include the orogenic periods (Fig. 6) and may also be consistent with ecological expansion of some *Nomocharis* species into new elevational ranges (Fig. 7e).

## Conclusions

*Lilium-Nomocharis* exhibits complex phylogenetic relationships typical of a pattern in which QTP and H-D Mountains endemic, morphologically and ecologically distinct vascular plant groups such as *Nomocharis*, are included within widespread ones, such as *Lilium*. Our phylogenetic results show that *Nomocharis* itself is paraphyletic and includes some species traditionally classified as *Lilium*; here, the N-N clade. Species of the N-N clade exhibit typical *Lilium* morphology, which distinguishes them from the *Nomocharis* species. Features characteristic of *Nomocharis*, such as horizon oriented and flat/open flowers are probably ancestral to the group, and evolved before the uplift of the QTP. However, such features may have enabled the invasion of the QTP and, later, the H-D Mountains by *Nomocharis* and should be the subject of future studies. Despite their differences, *Nomocharis* and the N-N clade have probably evolved some similarities due to differently timed expansions into diverse elevational habitats. Our phylogenetic results also show some circumstantial evidence for hybridization in among traditional *Lilium* and *Nomocharis* species, and that may help to explain the complex phylogenetic relationships within the *Lilium-Nomocharis* complex.

## Methods

### Plant materials

We reconstructed a molecular phylogeny of *Lilium* and *Nomocharis* using nuclear ITS and 294 total accessions, of which 67 were obtained from GenBank, 227 were collected with necessary permissions by the author, of which 30 were newly sequenced for this study (Table 2, Additional file 8: Table S1). Note that only 90 accessions used for our phylogenetic reconstruction have been sequenced for all plastid markers and ITS (Table 2, Additional file 8: Table S1). For molecular phylogenetic reconstructions of plastid DNA, we focused our sampling efforts on *Nomocharis* and its *Lilium* allies; namely *Lilium* species that are geographically and/or evolutionarily close to *Nomocharis*. Of particular note, we sampled *L. henrici* Franchet, *L. xanthellum* F. T. Wang & T. Tang, *L. saccatum* S. Y. Liang that are endemic to the H-D Mountains and have been sparsely sampled in previous studies. Among *Nomochari*s species, only *N. synaptica* Sealy, which is native to India, was not sampled. Additionally, we included representative species of *Lilium* from across the geographic and phylogenetic distribution of the genus. Altogether, for the plastid phylogeny we sampled 14 *Nomocharis* accessions representing seven of eight species, thirteen *Lilium* species for their geographic or evolutionary proximity to *Nomocharis*, and 29 additional *Lilium* species (Table 2). We selected representative accessions of other genera from within the Lilieae tribe as outgroups including two each of *Notholirion*, *Cardiocrinum* and *Fritillaria* (see [44]). Of the total 360 sequences that we used in this study, two hundred and sixty-five are new to our study, and these have collection, voucher, and Genbank accession information provided in Table 2. We have deposited downstream sequencing data, namely alignments and phylogenetic trees, in TreeBase (Submission number: 17567).

**Table 2:** Materials and GenBank accession numbers of five chloroplast makers and accession information

					Genbank accession numbers (bold indicated contributed by this study)				
Taxon name	Voucher(SZ)	GPS coordinates	Distribution	Abbreviation of taxa	*mat*K	*rbc*L	*trn*L-*trn*F	*rpl*32-*trn*L	*psb*A-*trn*H
*Lilium amabile* Palibin	G09017	N45°14′1.75″, E124°43′21″	C	LAM	*KF850798*	*KF850875*	*KF850981*	*KF850909*	*KF850830*
*Lilium anhuiense* D. C. Zhang & J. Z. Shao	G09001	N30°0′13.51″, E117°32′55″	C	LAH	*KF850803*	*KF850880*	*KF850994*	*KF850922*	*KF850835*
*Lilium bakerianum* Collett & Hemsley var. *rubrum* Stearn	G09008	N24°58′29″, E102°36′38″	B	LBKR1	HQ692243	HQ692342	*KF851009*	*KF850937*	HQ692442
*Lilium bakerianum* Collett & Hemsley var. *rubrum* Stearn	G09010	N26°23′10″, E102°47′15″	B	LBKR2	HQ692244	HQ692343	*KF851010*	*KF850938*	HQ692443
*Lilium bakerianum* var. *bakerianum* Collett & Hemsley	LQQ200901	N29°38′12″, E102°07′29″	B	LBK	HQ687300	HQ687318	*HQ687354*	*HQ687336*	KF850837
*Lilium brownii* var. *viridulum* Baker	G08031	N34°20′42″, E106°00′42″	BC	LBW	HQ692218	HQ692317	*KF850992*	*KF850920*	HQ692417
*Lilium cernuum* Komarov	G09018	N45°14′1″, E124°43′21″	C	LCM	*KF850799*	*KF850876*	*KF850982*	*KF850910*	*KF850831*
*Lilium concolor* Salisbury var. *pulchellum* (Fischer) Regel	G09012	N42°13′14″, E124°17′07″	BC	LCN	JN785993	JN786053	*KF850983*	*KF850911*	JN786023
*Lilium davidii* Duchartre ex Elwes	G2010062901	N29°03′37″, E107°12′07″	BC	LDV	HQ692179	HQ692279	*KF850986*	*KF850914*	HQ692378
*Lilium distichum* Nakai ex Kamibayashi	G09013	N42°14′28″, E127°25′11″	C	LDST	JN785999	JN786059	*KF850989*	*KF850917*	JN786029
*Lilium duchartrei* Franchet	G08018	N33°03′39″, E104°41′34″	B	LDC	*KF850807*	*KF850884*	*KF851018*	*KF850946*	*KF850841*
*Lilium fargesii* Franchet	G09011	N34°00′29″, E107°47′28″	B	LFG1	HQ687301	HQ687319	*HQ687355*	*HQ687337*	JN786032
*Lilium fargesii* Franchet	G2011015	N32°39′30″, E106°32′50″	B	LFG2	JN786006	JN786066	*KF851035*	*KF850963*	JN786036
*Lilium fargesii* Franchet	G2011016	N32°41′47″, E106°32′24″	B	LFG3	JN786007	JN786067	*KF851036*	*KF850964*	JN786037
*Lilium henrici* var. *henrici* Franchet	G09054	N27°47′10″, E98°32′42″	B	LHER	HQ687305	HQ687323	*HQ687359*	*HQ687341*	*KF850850*
*Lilium henryi* Baker	G08042	N27°21′15″, E106°13′55″	C	LHR	*KF850804*	*KF850881*	*KF851002*	*KF850930*	*KF850836*
*Lilium jinfushanense* L. J. Peng & B. N. Wang	G2010004	N29°01′54″, E107°11′18″	C	LJF1	HQ692257	HQ692356	*KF851007*	*KF850935*	HQ692456
*Lilium jinfushanense* L. J. Peng & B. N. Wang	G2010005	N29°02′18″, E107°12′37″	C	LJF2	HQ692258	HQ692357	*KF851008*	*KF850936*	HQ692457
*Lilium lankongense* Franchet	G2010082301-2	N27°47′07″, E99°38′42″	B	LLK1	HQ692247	HQ692346	*KF851012*	*KF850940*	HQ692446
*Lilium lankongense* Franchet	G2010071201-1	N27°07′35″, E100°14′31″	B	LLK2	HQ692248	HQ692347	*KF851013*	*KF850941*	HQ692447
*Lilium lankongense* Franchet	G2011007	N27°47′22″, E98°35′51″	B	LLK3	*KF850828*	*KF850905*	*KF851049*	*KF850977*	*KF850873*
*Lilium leucanthum* (Baker) Baker var. *centifolium* (Stapf ex Elwes) Stearn	Z0647	N30°32′37″, E104°17′33″	BC	LLUC	HQ692231	HQ692330	*KF851015*	*KF850943*	HQ692430
*Lilium leucanthum* (Baker) Baker var. *leucanthum*	G08030	N33°03′20″, E104°40′14″	BC	LLUE	HQ692230	HQ692329	*KF851014*	*KF850942*	HQ692429
*Lilium lijiangense* L. J. Peng	G09005	N26°21′44″, E102°48′45″	B	LLJ	*KF850805*	*KF850882*	*KF851006*	*KF850934*	*KF850838*
*Lilium longiflorum* Thunberg var. *scabrum* Masamune	Z05100	N26°21′44″, E102°48′45″	C	LLG	*KF850802*	*KF850879*	*KF850993*	*KF850921*	*KF850834*
*Lilium lophophorum* (Bureau & Franchet) Franchet	G08034	N30°52′05″, E108°52′01″	B	LLP1	HQ692196	HQ692296	*KF851021*	*KF850949*	HQ692395
*Lilium lophophorum* (Bureau & Franchet) Franchet	G2010081001-1	N29°08′32″, E100°04′50″	B	LLP2	HQ687303	HQ687321	*HQ687357*	*HQ687339*	HQ692403
*Lilium martagon* L. var. *pilosiusculum* Freyn	Em003	N46°44′49″, E84°25′57″	C	LMAT	*KF850801*	*KF850878*	*KF850988*	*KF850916*	*KF850833*
*Lilium matangense* J. M. Xu	G07009	N31°56′56″, E102°38′10″	B	LMT	HQ687302	HQ687320	*HQ687356*	*HQ687338*	*KF850840*
*Lilium nanum* Klotzsch	STET712	N28°30′04″, E98°07′49″	B	LNM1	HQ687295	HQ687313	*HQ687349*	*HQ687331*	*KF850844*
*Lilium nanum* Klotzsch	G2011001	N29°46′22″, E95°40′52″	B	LNM2	JN786008	JN786068	*KF851037*	*KF850965*	JN786038
*Lilium nanum* Klotzsch	G2011002	N29°46′22″, E95°40′52″	B	LNM3	JN786009	JN786069	*KF851038*	*KF850966*	JN786039
*Lilium nanum* Klotzsch	G2011003	N29°46′22″, E95°40′52″	B	LNM4	JN786010	JN786070	*KF851039*	*KF850967*	JN786040
*Lilium nanum* var. *flavidum* (Rendle) Sealy	G2011009	N28°30′04″, E98°07′49″	B	LNF1	*KF850823*	*KF850900*	*KF851044*	*KF850972*	*KF850868*
*Lilium nanum* var. *flavidum* (Rendle) Sealy	G2011009	N28°30′04″, E98°07′49″	B	LNF2	*KF850824*	*KF850901*	*KF851045*	*KF850973*	*KF850869*
*Lilium nanum* var. *flavidum* (Rendle) Sealy	G2011009	N28°30′04″, E98°07′49″	B	LNF3	*KF850825*	*KF850902*	*KF851046*	*KF850974*	*KF850870*
*Lilium nepalense* D. Don	YY10080907	N28°50′54″, E85°20′06″	A	LNP	HQ687299	HQ687317	*HQ687353*	*HQ687335*	N/A
*Lilium paradoxum* Stearn	G2011010	N29°37′47″, E94°24′14″	B	LPD1	*KF850826*	*KF850903*	*KF851047*	*KF850975*	*KF850871*
*Lilium paradoxum* Stearn	G2011010	N29°37′47″, E94°24′14″	B	LPD2	*KF850827*	*KF850904*	*KF851048*	*KF850976*	*KF850872*
*Lilium primulinum* Baker var. *burmanicum* (Franchet) Stearn	G2010082801	N27°20′36″, E100°09′23″	B	LPRO1	HQ692238	HQ692337	*KF851003*	*KF850931*	HQ692437
*Lilium primulinum* Baker var. *ochraceum* (Franchet) Stearn	WZX2010090101	N27°01′20″, E100°13′24″	B	LPRO2	HQ692236	HQ692335	*KF851004*	*KF850932*	HQ692435
*Lilium primulinum* Baker var. *ochraceum* (Franchet) Stearn	G09009	N26°00′50″, E98°37′04″	B	LRPO3	HQ692240	HQ692339	*KF851005*	*KF850933*	HQ692439
*Lilium pumilum* Redouté	G08007	N35°47′49″, E104°03′49″	C	LPM1	HQ692180	HQ692280	*KF850979*	*KF850907*	HQ692379
*Lilium pumilum* Redouté	G08008	N35°47′56″, E104°03′06″	C	LPM2	HQ692181	HQ692281	*KF850980*	*KF850908*	HQ692380
*Lilium regale* E. H. Wilson	G09020	N31°29′38″, E103°36′49″	B	LRG1	HQ692192	HQ692292	*KF850995*	*KF850923*	HQ692391
*Lilium regale* E. H. Wilson	G07026	N31°30′23″, E103°33′29″	B	LRG2	HQ692191	HQ692291	*KF850996*	*KF850924*	HQ692390
*Lilium saccatum* S. Yun Liang	G2010070902	N29°37′47″, E94°24′14″	B	LSC1	HQ687297	HQ687315	*HQ687351*	*HQ687333*	*KF850845*
*Lilium saccatum* S. Yun Liang	STET1261	N29°46′22″, E95°40′52″	B	LSC2	HQ687298	HQ687316	*HQ687352*	*HQ687334*	*KF850846*
*Lilium sargentiae* E. H. Wilson	G08032	N29°04′37″, E107°12′08″	B	LSG1	HQ692214	HQ692313	*KF850997*	*KF850925*	HQ692413
*Lilium sargentiae* E. H. Wilson	G08006	N31°06′26″, E103°33′37″	B	LSG2	HQ692213	HQ692312	*KF850998*	*KF850926*	HQ692412
*Lilium sempervivoideum* H. Léveillé	G09006	N27°49′34″, E102°15′34″	B	LSMP	*KF850806*	*KF850883*	*KF851016*	*KF850944*	*KF850839*
*Lilium* sp.	G2010090302	N28°12′27″, 99°58′14″	B	LSOL1	*KF850808*	*KF850885*	*KF851019*	*KF850947*	*KF850842*
*Lilium* sp.	G2010081705	N28°08′27″, 99°18′15″	B	LSOL2	*KF850809*	*KF850886*	*KF851020*	*KF850948*	*KF850843*
*Lilium souliei* (Franchet) Sealy	G2011004	N28°30′04″, E98°07′49″	B	LSOL3	JN786012	JN786072	*KF851040*	*KF850968*	JN786042
*Lilium souliei* (Franchet) Sealy	STET713	N28°30′04″, E98°07′49″	B	LSOL4	JN786013	JN786073	*KF851041*	*KF850969*	JN786043
*Lilium speciosum* Thunberg var. *gloriosoides* Baker	G09032	N30°05′15″, E117°29′25″	C	LSP	*KF850797*	*KF850874*	*KF850978*	*KF850906*	*KF850829*
*Lilium sulphureum* Baker ex J. D. Hooker	G09028	N23°15′03″, E104°16′03″	B	LSL1	HQ692226	HQ692325	*KF850999*	*KF850927*	HQ692425
*Lilium sulphureum* Baker ex J. D. Hooker	G09029	N23°15′03″, E104°16′03″	B	LSL2	HQ692225	HQ692324	*KF851000*	*KF850928*	HQ692424
*Lilium sulphureum* Baker ex J. D. Hooker	G09030	N25°50′26″, E98°54′38″	B	LSL3	HQ692224	HQ692323	*KF851001*	*KF850929*	HQ692423
*Lilium taliense* Franchet	G2010071801	N28°04′10″, E99°46′29″	B	LTL	HQ692209	HQ692308	*KF851011*	*KF850939*	HQ692408
*Lilium tigrinum* Ker Gawler	Z0692	N31°48′40″, E104°26′51″	BC	LTG1	HQ692193	HQ692293	*KF850984*	*KF850912*	HQ692392
*Lilium tigrinum* Ker Gawler	G0833	N34°03′13″, E107°30′15″	BC	LTG2	HQ692195	HQ692295	*KF850985*	*KF850913*	HQ692394
*Lilium tsingtauense* Gilg.	G201101	N36°10′1″, E120°34′23″	C	LTS	*KF850800*	*KF850877*	*KF850987*	*KF850915*	*KF850832*
*Lilium wardii* Stapf ex F. C. Stern	G2011007	N29°58′21″, E95°21′48″	B	LWD1	JN786014	JN786074	*KF851042*	*KF850970*	JN786044
*Lilium wardii* Stapf ex F. C. Stern	G2011008	N29°57′43″, E 94°47′27″	B	LWD2	JN786015	JN786075	*KF851043*	*KF850971*	JN786045
*Lilium wenshanense* L. J. Peng & F. X. Li	G09002	N26°00′50″, E98°37′04″	B	LWS1	HQ692232	HQ692331	*KF850990*	*KF850918*	HQ692431
*Lilium wenshanense* L. J. Peng & F. X. Li	WJ10051401	N31°50′32″, E104°39′36″	B	LWS2	HQ692235	HQ692334	*KF850991*	*KF850919*	HQ692434
*Lilium xanthellum* F. T. Wang & Tang var. *luteum* S. Yun Liang	G2010070106-1	N29°02′39″, E99°42′41″	B	LXAL	HQ692255	HQ692354	*KF851017*	*KF850945*	HQ692454
*Lilium xanthellum* var. *xanthellum* F. T. Wang & Tang	G2010070106-2	N29°02′39″, E99°42′41″	B	LXA	HQ687304	HQ687322	*HQ687358*	*HQ687340*	HQ692451
*Lilium yapingense* Y. D. Gao et X. J. He	G2010070903	N27°12′20″, E98°44′24″	B	LYP	HQ687296	HQ687314	*HQ687350*	*HQ687332*	*KF850847*
*Nomocharis aperta* (Franchet) E. H. Wilson	Z0674	N27°47′41″, E99°54′27″	B	NAP7	HQ687306	HQ687324	*HQ687360*	*HQ687342*	*KF850853*
*Nomocharis aperta* (Franchet) E. H. Wilson	G10ZDNA01	N28°1′8″, E99°45′41″	B	NAP1	*KF850811*	*KF850888*	*KF851023*	*KF850951*	*KF850854*
*Nomocharis aperta* (Franchet) E. H. Wilson	G10ZDNA02	N27°31′14″, E99°52′43″	B	NAP2	*KF850812*	*KF850889*	*KF851024*	*KF850952*	*KF850855*
*Nomocharis aperta* (Franchet) E. H. Wilson	G10ZDNA03	N27°30′30″, E99°48′33″	B	NAP3	*KF850813*	*KF850890*	*KF851025*	*KF850953*	*KF850856*
*Nomocharis aperta* (Franchet) E. H. Wilson	G10ZDNA04	N27°26′33″, E99°48′33″	B	NAP4	*KF850814*	*KF850891*	*KF851026*	*KF850954*	*KF850857*
*Nomocharis aperta* (Franchet) E. H. Wilson	G10ZDNA05	N28°1′8″, E99°45′41″	B	NAP5	*KF850815*	*KF850892*	*KF851027*	*KF850955*	*KF850858*
*Nomocharis aperta* (Franchet) E. H. Wilson	G10ZDNA06	N28°1′8″, E99°45′41″	B	NAP6	*KF850816*	*KF850893*	*KF851028*	*KF850956*	*KF850859*
*Nomocharis basilissa* Farrer ex W. E. Evans	G2010070904	N27°12′20″, E98°44′24″	B	NBA	HQ687308	HQ687326	*HQ687362*	*HQ687344*	N/A
*Nomocharis farreri* (W. E. Evans) Harrow	G09037	N25°58′43″, E98°40′20″	B	NFR	HQ687309	HQ687327	*HQ687363*	*HQ687345*	*KF850860*
*Nomocharis gongshanensis* Y. D. Gao et X. J. He	G09003	N27°46′09″, E98°26′58″	B	NGS	*KF850810*	*KF850887*	*KF851022*	*KF850950*	*KF850848*
*Nomocharis meleagrina* Franchet	G09038	N27°46′18″, E98°27′20″	B	NML	HQ687310	HQ687328	*HQ687364*	*HQ687346*	*KF850861*
*Nomocharis pardanthina* f. *punctulata* Sealy	G09040	N27°46′09″, E98°26′58″	B	NPDF	HQ687307	HQ687325	*HQ687361*	*HQ687343*	*KF850852*
*Nomocharis pardanthina* Franchet	G09036	N25°42′28″, E100°06′27″	B	NPD	HQ687311	HQ687329	*HQ687365*	*HQ687347*	*KF850851*
*Nomocharis saluenensis* I. B. Balfour	G09039	N27°46′13″, E98°26′44″	B	NSL	HQ687312	HQ687330	*HQ687366*	*HQ687348*	*KF850849*
*Cardiocrinum cathayanum* (E. H. Wilson) Stearn	G09045	N30°04′10″, E117°48′11″	C	/	*KF850819*	*KF850896*	*KF851031*	*KF850959*	*KF850864*
*Cardiocrinum giganteum* (Wallich) Makino	Z05023	N29°02′18″, E107°12′37″	B	/	*KF850820*	*KF850897*	*KF851032*	*KF850960*	*KF850865*
*Fritillaria cirrhosa* D. Don	G09048	N27°19′40″, E102°27′44″	B	/	*KF850818*	*KF850895*	*KF851030*	*KF850958*	*KF850863*
*Fritillaria thunbergii* Miquel	G09100	N32°6′2″, E118°56′27″	C	/	*KF850817*	*KF850894*	*KF851029*	*KF850957*	*KF850862*
*Notholirion bulbuliferum* (Lingelsheim ex H. Limpricht) Stearn	G07002	N31°45′43″, E102°15′35″	B	/	*KF850822*	*KF850899*	*KF851034*	*KF850962*	*KF850867*
*Notholirion macrophyllum* (D. Don) Boissier	G09043	N29°2′34.77″, E100°32′30.01″	AB	/	*KF850821*	*KF850898*	*KF851033*	*KF850961*	*KF850866*

We surveyed the morphology of *Nomocharis*, its close allies, and major lineages throughout *Lilium*. In particular, we used photographs of specimens observed in the field, field collected materials, and greenhouse specimens to assess macromorphological traits of 14 species of *Nomocharis* and closely related species of *Lilium*. To evaluate the same characters more broadly in 10 major lineages of *Lilium* (based on our phylogenetic results) we examined preserved specimens available to us, utilized the Chinese Virtual Herbarium, and obtained data from the literature (e.g., Flora of China [20]).

### DNA extraction, Polymerase Chain Reaction (PCR) and sequencing

We selected the nuclear marker ITS and the cpDNA regions *trn*L-F, *rbc*L, *mat*K, *rpl*32-*trn*L and *psb*A-*trn*H to reconstruct the molecular phylogeny of *Lilium*-*Nomocharis*. We chose the five cpDNA makers because three of them have been proposed as DNA barcodes for their high resolution and amplification success [45], and the other two have shown suitable variation in preliminary analyses (data not shown). For PCR amplifications of nuclear and plastid markers, we used total DNA extractions from fresh or silica gel-dried leaf tissue using a modified cetyltrimethyl-ammonium bromide (CTAB) protocol by Doyle and Doyle [46] or the Plant Genomic DNA Kit (TIANGEN Biotech, Beijing, China). We amplified all six markers using the primers listed in Table 3. All PCR reactions were performed with 50 ng genomic DNA in 20 μl reactions in a GeneAmp PCR System 9700 (Applied Biosystems, USA). The ITS reactions were performed using the following thermocycler protocol: 94 °C denaturation for 2 min; 35 cycles of 94 °C denaturation for 30 s, 55 °C primer annealing for 30 s, and 72 °C extension for 60 s; and a final extension of 72 °C for 10 min. For the plastid markers, the amplification conditions were the same except that primer annealing was performed at 52 °C for 45 s each cycle. Our amplified PCR products were sent to Invitrogen Biotech Co. Ltd. (Shanghai, China) for purification and sequencing, which was done on an ABI-3730XL DNA sequencer. For each sequenced accession, forward and reverse sequencing reactions were performed for increased coverage. Sequencing of the *psb*A-*trn*H spacer failed in two species, *Nomocharis basilissa* and *Lilium nepalense*, due to homopolymers at ~200 bp from the 5’ end. Thus, all data for this marker for these two species was considered missing (i.e., '?’, [47]) in downstream phylogenetic analyses.

**Table 3:** Primers and sequences statistics of nuclear and chloroplast makers used in present study

Region	Forward-primer (5′-3′)	Reverse-primer (5′-3′)	Reference	Alignment length (bp)	Variable sites	Parsimony informative sites
ITS	GGAAGTAAAAGTCGTAACAAGG	TCCTCCGCTTATTGATATGC	[92]	673	398	287
*rbc*L	ATGTCACCACAAACAGAGAC	TCACATGTACCCGCAGTAGC	[93]	796	84	42
*mat*K	CGATCTATTCATTCAATATTTC	TCTAGCACACGAAAGTCGAAGT	[94]	392	33	23
*trn*L intron and *trn*L-*trn*F spacer	CGAAATCGGTAGACGCTACG	ATTTGAACTGGTGACACGAG	[95]	786	57	34
*rpl*32-*trn*L(UAG)	CAGTTCCAAAAAAACGTACTTC	CTGCTTCCTAAGAGCAGCGT	[45]	842	138	100
*psb*A-*trn*H	ACTGCCTTGATCCACTTGGC	CGAAGCTCCATCTACAAATGG	[96]	613	24	19
			Total plastid	3429	336	218

### Molecular analysis

We aligned our DNA sequences using ClustalX [48] and then by eye in MEGA4.0 [49] following the guidelines of Morrison [50]. We trimmed the sequences to the limits of the ITS and the plastid regions, respectively, by comparing with examples deposited in Genbank. We positioned gaps to minimize nucleotide mismatches. We combined the five cpDNA markers into a single dataset, and all six aligned, and curated datasets were used to calculate uncorrected pairwise nucleotide differences in PAUP* version 4.0b10 [51]. Our nuclear ITS dataset contained a total of 294 accessions, inclusive of our eight outgroups. The ITS matrix contained 673 characters of which 398 were variable and 271 were parsimony-informative. There were 90 accessions for which sequences of all chloroplast markers were available, including for six outgroups. Details of the five chloroplast makers are presented in Table 3. The combined cpDNA alignment was 3429 bp long and contained 336 variables sites, of which 218 (or 6.3 %) were parsimony informative.

For phylogenetic analyses, we combined all five plastid sequences, because chloroplast genes have shared evolutionary histories within the chloroplast genome and because they do not recombine. We treated the ITS dataset independently. Bayesian phylogenetic analyses of the combined chloroplast dataset and the ITS dataset were conducted using MrBayes version 3.1.2 [52] with the GTR+ G + I and GTR+ G models of nucleotide substitution, respectively. These models were selected under the Akaike information criterion (AIC) using MrModeltest version 2.2 [53]. For each of the two datasets, we performed two simultaneous Bayesian analyses that started from a random tree and ran for 10 million generations with sampling every 1000 generations. Within each simultaneous run, four independent MCMC chains were used and the temperature increment between chains was adjusted to 0.2 based on mixing observed in preliminary analyses. Variation in likelihood scores was examined graphically for each independent run using Tracer 1.4 [54] and was used to determine apparent stationarity. Based on observations in Tracer, the first 25 % (2500) of posterior trees were discarded from each run as “burn-in” and posterior probabilities (pp) of clades were calculated from the remaining trees. Following burnin, we selected the best tree from among the simultaneous analyses of the plastid and ITS dataset, independently, using maximum clade credibility.

Maximum parsimony (MP) analyses of the ITS and the combined chloroplast makers were carried out using PAUP* [51]. Characters were treated as unordered and unweighted. A heuristic search was performed with 1000 replicate analyses, random stepwise addition of taxa, tree-bisection-reconnection (TBR) branch swapping, and maximum trees set to 50,000. We summarized the resulting equally parsimonious topologies using majority-rule consensus and calculated bootstrap values from one million replicate analyses using fast stepwise addition of taxa. We retained the bootstrap values for clades consistent with the majority-rule consensus tree.

We carried out topological testing using Kishino-Hasegawa (KH) tests in PAUP*, because KH tests are known to exhibit very low type I error rates [55]. To perform the tests, we used a reduced dataset, which consisted of one sequence for each major evolutionary lineage that was mutually represented in the plastid and nuclear gene trees (Additional file 7: Figure S7). We confirmed that the selected samples produced the same arrangements of evolutionary lineages as the entire plastid and nuclear alignments by generating maximum likelihood (ML) trees using the GTR+ G + I and GTR+ G models, respectively (data not shown). Major lineages were manually organized into plastid and nuclear cladograms in Mesquite [56] (Additional file 7: Figure S7). The reduced alignments plus the cladograms were loaded into PAUP* for performing the KH tests. Specifically, we used the tests to determine if each tree represented a significantly better fit for the dataset from which it was reconstructed compared to tree resulting from the other dataset. We performed the KH tests under the GTR+ G + I and GTR+ G models for the plastid and nuclear datasets using a normal test distribution.

### Statistical parsimony network

We expected that strictly bifurcating trees may not completely describe the evolutionary relationships within *Lilium-Nomocharis*, because hybridization in *Lilium-Nomocharis* has been postulated [13, 17, 57] and incomplete lineage sorting has been detected in many plant lineages [40]. Therefore, we used the statistical parsimony network approach implemented in TCS v.1.21 [58] to further evaluate evolutionary relationships within the *Lilium-Nomocharis* complex using the combined chloroplast sequences. We built the parsimony network using eighty-four accessions sequenced for all cpDNA markers except *psb*A-*trn*H, which was missing data for two taxa (see above). We tested whether removal of *psb*A-*trn*H would change relationships among species, by reconstructing a bifurcating plastid phylogeny without the marker, and it showed no differences compared to the tree constructed using whole dataset (results not shown). For the network analysis, we considered each indel as a single mutation event, and all indels were reduced to single characters (arbitrarily A or T) in a final alignment. The resulting plastid matrix was 3037 characters in length and contained 66 plastid haplotypes representing 84 accessions of *Lilium*-*Nomocharis*. We eliminated loops from the parsimony based on the principle that haplotypes with interior positions in the network are assumed to be ancestral [59].

### Divergence estimation

Molecular dating in Liliales has been previously performed using distantly related fossils [60], calibrations from previous studies [44, 61], and single calibration points [17]. In particular, Bremer [60] dated nodes in the monocot phylogeny using fossils closely related to palms, aroids, grasses, and cattails and found that Liliales evolved approximately 112 Mya and began diversifying 82 ± 10 Mya. Deriving calibration points from Bremer [60], Patterson and Givnish [44] inferred the divergence time of the tribe Lilieae as 12 Mya and Vinnersten and Bremer [61] concluded that the monophyletic lineage comprised of *Lilium*, *Nomocharis* and *Fritillaria* diverged 6 ± 2.9 Ma. Gao et al. [17] provided a detailed review of Liliales fossils and performed dating using a single, reliable fossil of *Smilax*, *Smilax wilcoxensis* Berry [62], to calibrate the divergence between Liliaceae and Smilaceae. Their results showed that Lilieae evolved approximately 16mya. Despite these efforts, it has been widely discussed and shown that single calibration points and caibrations derived from prior studies lead to less reliable, and often younger, clade ages [63–65].

We sought to more rigorously date events in *Lilium*-*Nomocharis* by applying two calibration points for dating analyses in BEAST (Additional file 2: Figure S2) [66, 67]. For one calibration, we constrained the divergence time of Liliaceae and Smilacaceae using *Smilax wilcoxensis*. In brief, *Smilax wilcoxensis* is known from the early Eocene (∼48.6–55.8 Mya) of the Tennessee Wilcox Formation [62, 68], which is assigned a relative age based on pollen [69, 70]. Specifically, we calibrated the Liliaceae-Smilacaceae node using a uniform prior with a lower bound (paleontologically upper) of 48.6 Mya and an upper bound of 131 Mya. Thus, we asserted our belief that Smilacaceae cannot be younger than *Smilax wilcoxensis* or older than the Barremian (i.e., 131 Mya), from which the oldest flowering plant fossil is known [71]. For the second calibration, we used *Ripogonum tasmanicum* Conran, et al. [72] to constrain the age of the ancestor of the monotypic Ripogonanceae and Philesiaceae (following Angiosperm Phylogeny Website, [73]). *Ripogonum tasmanicum* is reported from the Tasmanian Macquarie Harbour Formation [72], which is approximately 51–52 million years old based on a foraminiferal index [74]. Thus, we constrained the Ripogonanceae and Philesiaceae split using a uniform prior with a lower bound of 51 Mya and an upper bound of 131 Mya. The prior asserts our belief that Ripogonaceae cannot be younger than its fossil or older than the earliest known flowering plant.

The two fossils facilitated establishing calibration points that were well outside of the *Nomocharis*-*Lilium* complex. Therefore, we applied these two calibrations to infer the split between *Lilium* and *Fritillaria* using a dataset comprised of three cpDNA markers (*apt*F-H, *mat*K and *rbc*L, see Additional file 9: Table S2, Additional file 2: Figure S2) that included 45 representative Liliales species and more than 3000 bp [75]. We applied the result mean and 95 % Highest Posterior Density (HPD) to constrain the *Lilium* and *Fritillaria* node using a normal prior distribution in an analysis of our plastid dataset. We take these results (Additional file 2: Figure S2) to be our best estimates of ages within *Lilium-Nomocharis*. More vetted fossils closer to *Lilium* may eliminate the need for the second dating step in the future.

Divergence time estimations were performed using BEAST ver. 1.5.3 [67] identically for the *cp*DNA and ITS datasets. The normal prior distribution on the age of the *Lilium* stem node (i.e., the split of *Lilium* and *Fritillaria*) was set using a mean of 14.92 Mya and a standard deviation of 2.5. The chosen standard deviation gave a 95 % HPD of 10.81-19.03 Ma, which was slightly narrower than the actual result of 6.32–25.71 Ma. A likelihood ratio test in PAUP 4.10b [51] rejected strict clocks for both datasets (*P* < 0.01), therefore we used an uncorrelated lognormal (UCLN), relaxed clock [76]. We used the GTR + G + I and GTR + G models of nucleotide substitution for combined plastid and nuclear ITS dataset, respectively. For the distribution of divergence times, a pure birth branching process (Yule model) was chosen as a prior. BEAST analyses were run on the Cyberinfrastructure for Phylogenetic Research (CIPRES) Science Gateway (http://www.phylo.org/portal2). We ran two independent Markov chains, each for 50,000,000 generations, initiated with a random starting tree, and sampled every 1000 generations. The first 20 % of sampled trees from all runs were discarded as burn-in based on visual inspection in Tracer version 1.4 [54].

### Ancestral Area Reconstructions (AAR)

We used the Bayesian Binary method (BBM) in Reconstruct Ancestral States in Phylogenies 2.1b (RASP 2.0) [77–79] to reconstruct the biogeographic history of *Lilium*-*Nomocharis* on the ITS consensus phylogeny constructed from BEAST trees. Based on prior studies (e.g., [20, 80]) three areas of endemism were recognized: Qinghai-Tibetan Plateau (QTP, A), H-D Mountains (HDM, B), the geographic region now covered by Sino-Japanese Forest subkingdom (SJFS, C; A-C stand for each region in the RASP analyses, Table 2). We compared BBM results to results from Lagrange, which implements a likelihood method and the Dispersal-Extinction-Cladogenesis (DEC) model [81]. In Lagrange, we set migration probabilities among the three areas of endemism to 1.0 throughout time and did not limit the number of areas that a widespread taxon could occupy (Additional file 10: Table S3). We allowed Lagrange to estimate the extinction and dispersal parameters required for the DEC model.

### Ancestral state reconstruction (ASR)

We reconstructed the ancestral states for four, variable macro morphological characters and the habitat characteristic, elevation, in *Lilium*-*Nomocharis*. We selected variable macromorphological characters with states that could be evaluated with confidence given the coarse availability of specimen data (see *Taxon sampling* above). Specifically, we performed reconstructions for corolla shape, flower orientation, the ratio of stigma versus stamen length, and leaf arrangement (Additional file 11: Table S4). We selected these characters from among other plausible ones, because they have previously been used to delimit species within *Lilium* and *Nomocharis* [19, 20, 80] but they have not been previously considered within a phylogenetic framework. For corolla shape, we coded species as having flat or open flowers, campaniform or bell shaped flowers, recurved, funnel or trumpet shaped, or bowl-shaped. Flower orientation states were coded as nodding, horizon, and up (i.e., upward facing). For stigma-stamen ratio, we coded states as being greater than 1.25, less than 0.75, or between 0.75 and 1.25. Using these ranges for stigma-stamen ratios enabled us to code species visually. Leaf arrangement was coded as being alternate or whorled. The whorled leaf character was assigned to species that have 3+ leaves arising from a single node and species with scattered leaves arising asynchronously [82]. For elevation, we acquired information from floras and specimen records on GBIF (http://www.gbif.org/). We treated elevation as categorical by using 1000 ft. increments for our discrete character states.

To reconstruct the ancestral character states we used BBM in RASP, which is not limited to historical biogeographic applications. We performed the reconstructions of ancestral morphological states across the dated ITS consensus tree resulting from the BEAST analysis and using the character matrices presented in Additional file 9: Table S2. We modified the BEAST consensus tree using TreeGraph 2.0 [83] by pruning outgroups and collapsing the major clades except *Nomocharis*. We did this to avoid confounding the issue with outgroups, which were not completely sampled or studied, and to simplify the reconstructions for less well sampled clades outside of *Nomocharis*. Branch length and divergence time information were preserved. The Bayesian analyses in RASP were carried out using default settings except that we ran the analyses for 1,000,000 MCMC generations and used the F81 + G model for changes between states.

## Additional files

Additional file 1: Figure S1. Reconstructed phylogenetic relationship of whole *Lilium-Nomocharis* based on Bayesian inferences of ITS dataset. Names of terminal clades based on Comber [23] and Liang [19]. The Sinomartagon I clade is highlighted. Additional file 2: Figure S2. Divergence dating of major clades of Liliales using two fossil calibrations (1 and 2). Additional file 3: Figure S3. Pictures from western China showing: a-c *Lilium henrici* var. *henrici*; d-f *L. lophophorum*; g-i *L. saccatum*; j-l *L. yapingense*. Additional file 4: Figure S4. Pictures from western China showing: a-e *Lilium xanthellum* with variations on tepal morphology within a same locality; g-i, flower of *L. souilei*, *L. nanum* and *L. nepalense*; j-l, habit of *L. souilei*, *L. nanum* and *L. nepalense*. Additional file 5: Figure S5. Pictures from western China showing *Nomocharis*: a-c, *N. pardanthina*; d-f, *N. saluenensis*; g-i, *N. pardanthina* f. *punctulata*. Additional file 6: Figure S6. Outer and inner tepals comparison in a, *Lilium henrici*; b, *L. lophophorum*; c-d, two types of *L. xanthellum*; e, *L. yapingense*; f, *L. saccatum*; g, *Nomocharis saluenensis*; h, *N. pardanthina* f. *punctulata*; i-j, two types of *N. aperta* (Zhongdian and Fugong, respectively); k, *N. basilissa*; l, *N. gongshanensis*; m, *N. pardanthina*; n, *N. meleagrina*. Additional file 7: Figure S7. Results of KH tests for ITS and combined plastid datasets. Additional file 8: Table S1. Sources of ITS sequence data. Additional file 9: Table S2. Genbank accessions used in diversification dating of major clades of Liliales. Additional file 10: Table S3. The matrix of model used in AAR analysis of LARANGE. Additional file 11: Table S4. Morphological character states used in ancestral state reconstruction.
