# Supplementary figures and images for: Morphological and ecological divergence of Lilium and Nomocharis within the Hengduan Mountains and Qinghai-Tibetan Plateau may result from habitat specialization and hybridization
Source: BMC Evol Biol. 2015 Jul 29;15:147. doi: 10.1186/s12862-015-0405-2 (PMC4518642; doi:10.1186/s12862-015-0405-2)

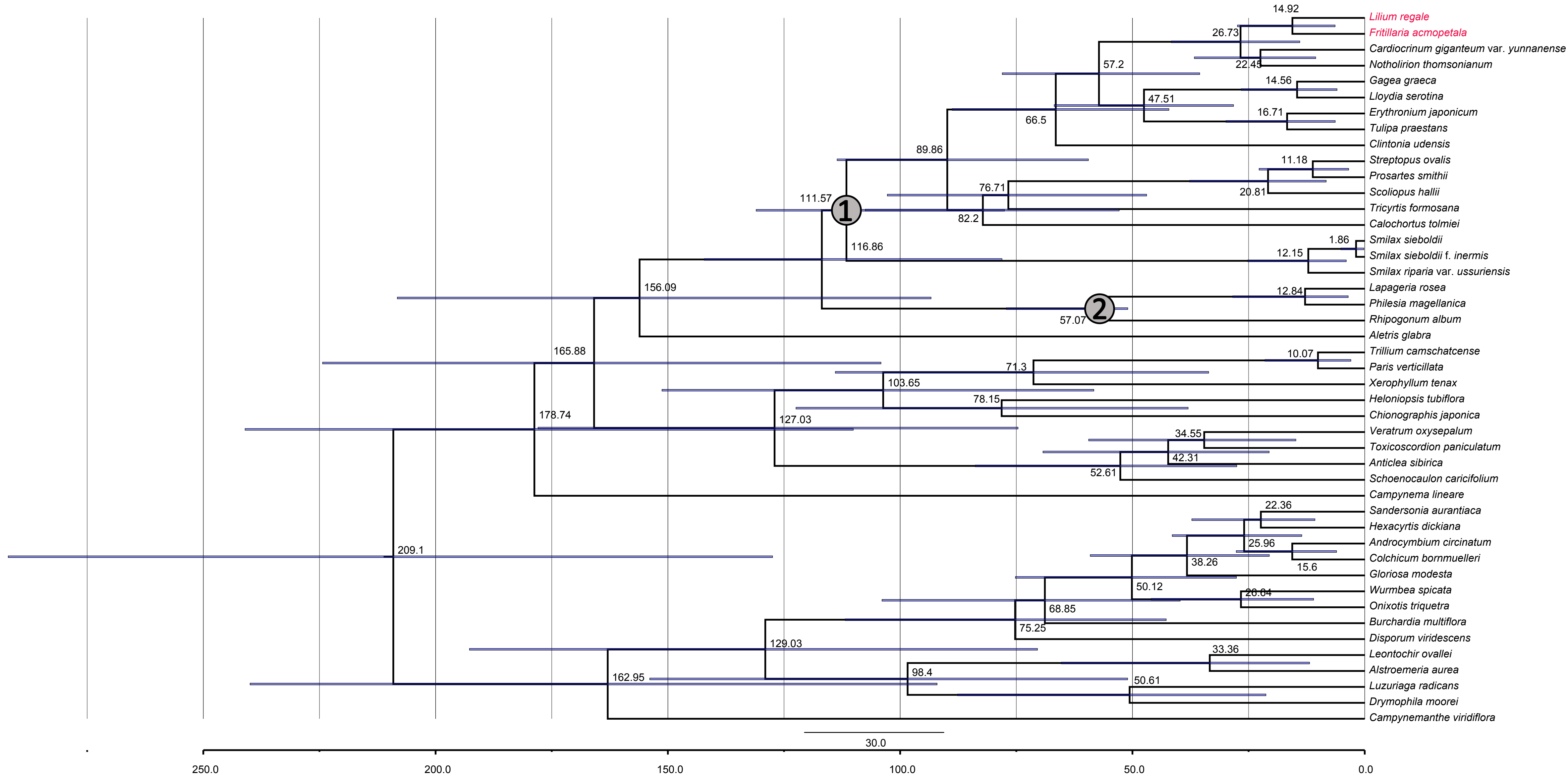

Supplement: Additional file 2: Figure S2. — Divergence dating of major clades of Liliales using two fossil calibrations (1 and 2). [file 12862_2015_405_MOESM2_ESM.pdf]

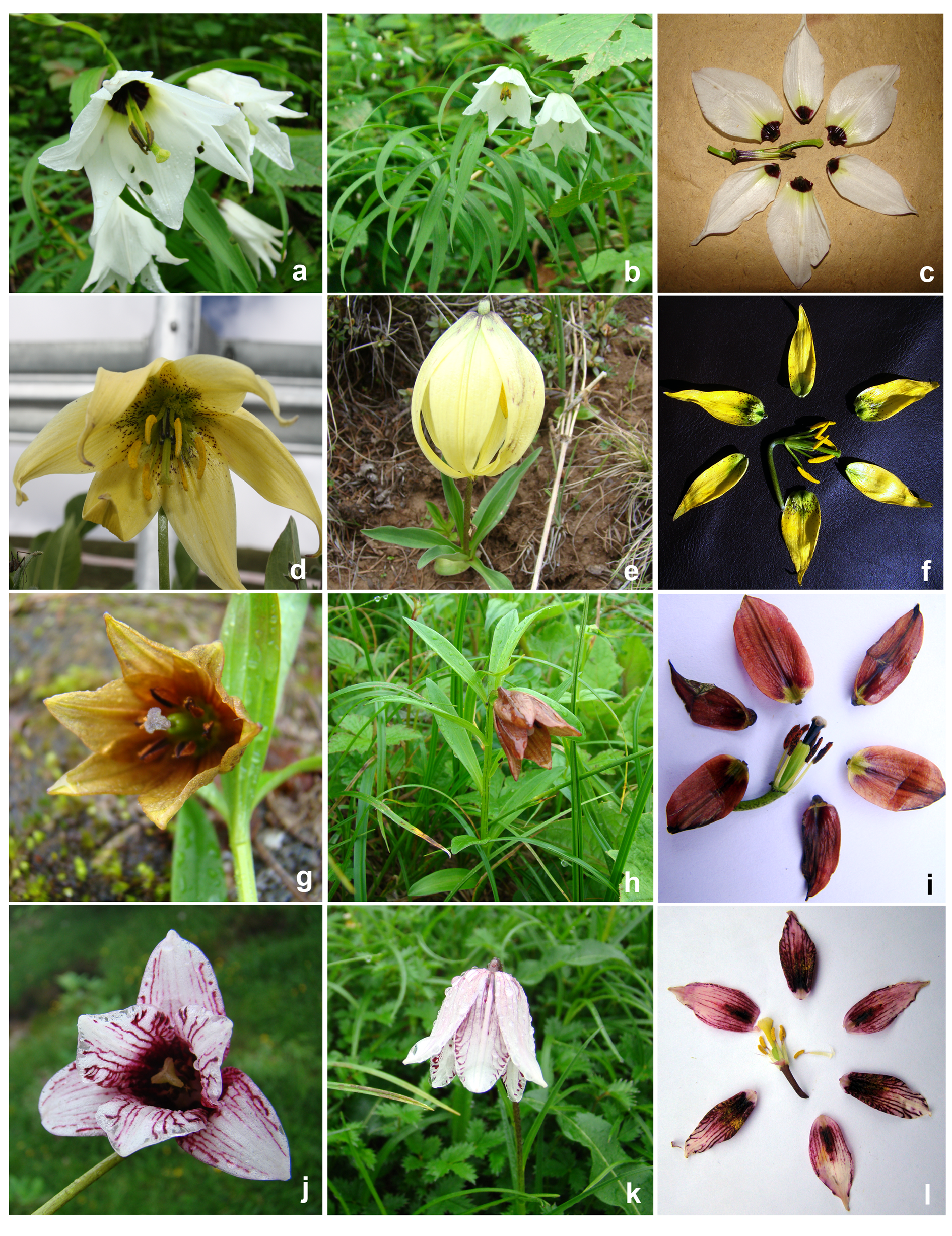

Supplement: Additional file 3: Figure S3. — Pictures from western China showing: a-c Lilium henrici var. henrici; d-f L. lophophorum; g-i L. saccatum; j-l L. yapingense. [file 12862_2015_405_MOESM3_ESM.tif]

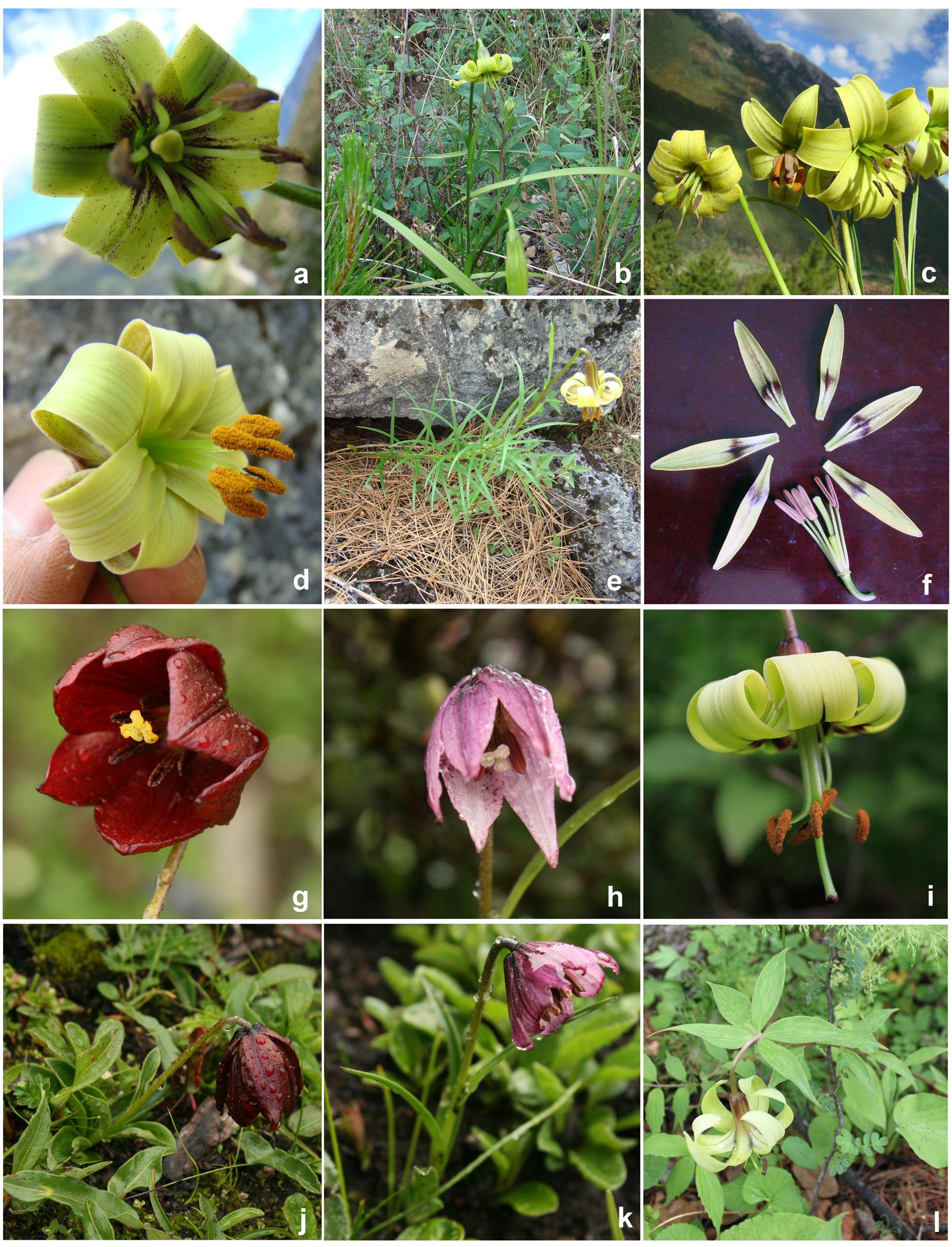

Supplement: Additional file 4: Figure S4. — Pictures from western China showing: a-e Lilium xanthellum with variations on tepal morphology within a same locality; g-i, flower of L. souilei, L. nanum and L. nepalense; j-l, habit of L. souilei, L. nanum and L. nepalense. [file 12862_2015_405_MOESM4_ESM.tif]

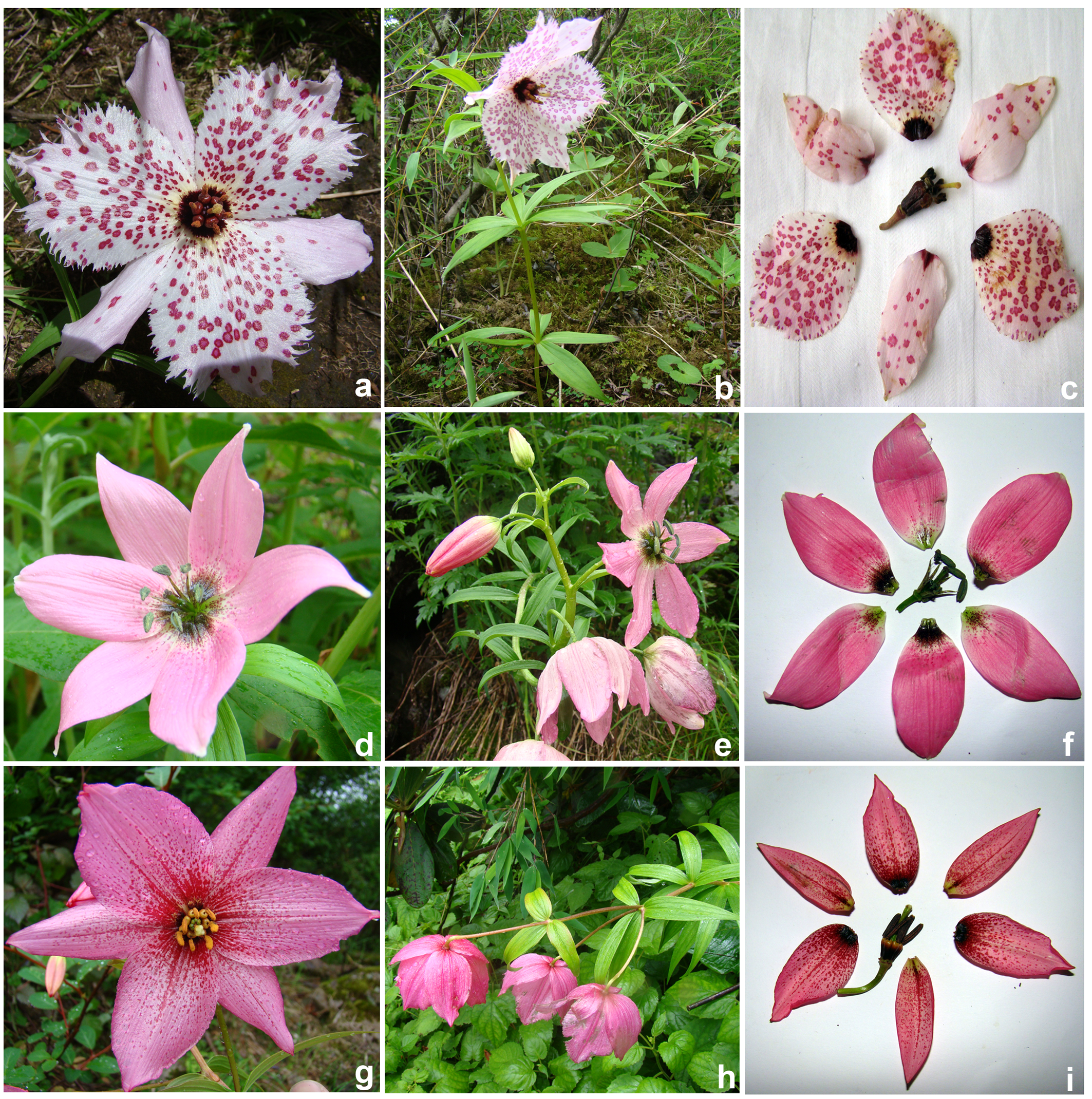

Supplement: Additional file 5: Figure S5. — Pictures from western China showing Nomocharis: a-c, N. pardanthina; d-f, N. saluenensis; g-i, N. pardanthina f. punctulata. [file 12862_2015_405_MOESM5_ESM.tif]

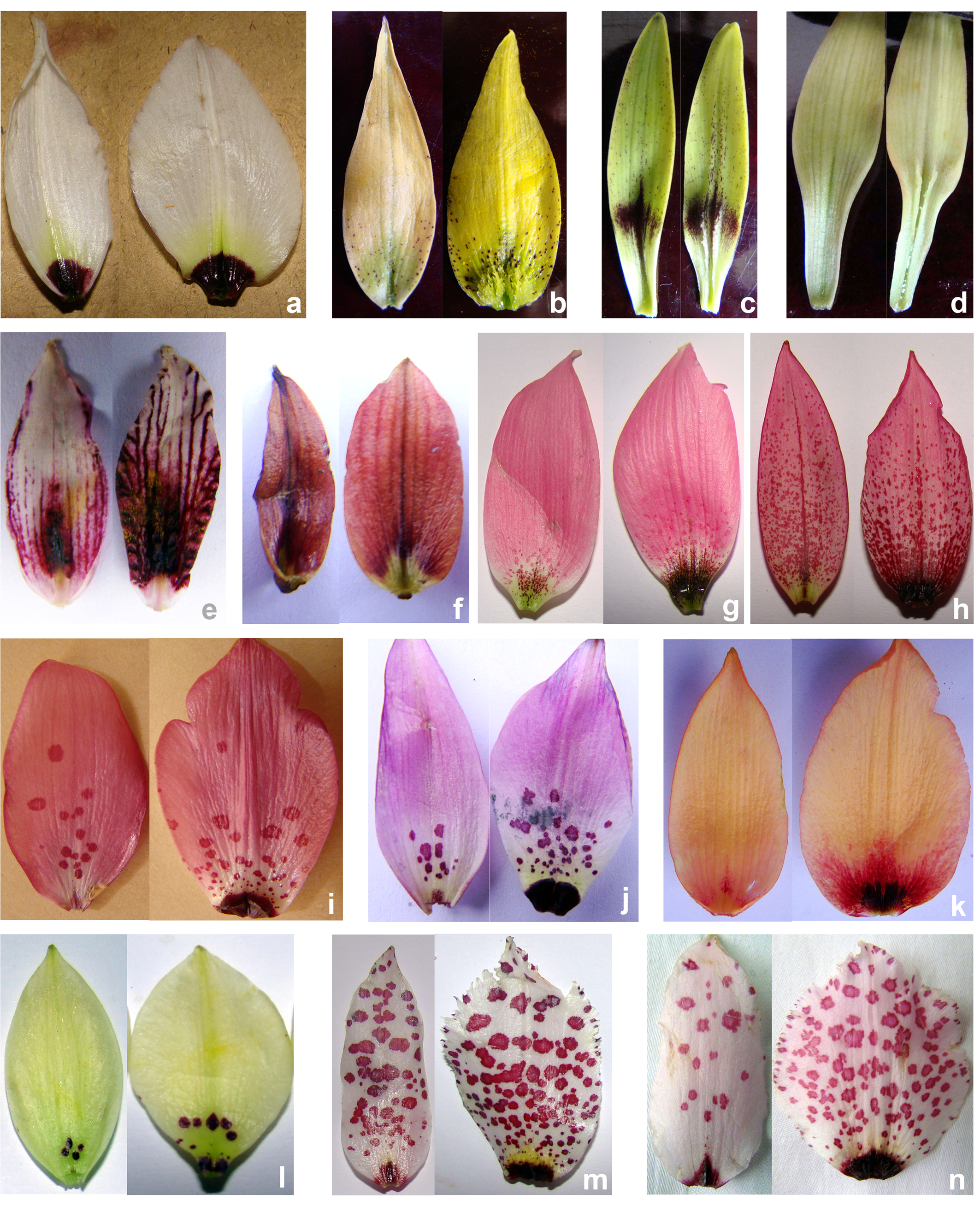

Supplement: Additional file 6: Figure S6. — Outer and inner tepals comparison in a, Lilium henrici; b, L. lophophorum; c-d, two types of L. xanthellum; e, L. yapingense; f, L. saccatum; g, Nomocharis saluenensis; h, N. pardanthina f. punctulata; i-j, two types of N. aperta (Zhongdian and Fugong, respectively); k, N. basilissa; l, N. gongshanensis; m, N. pardanthina; n, N. meleagrina. [file 12862_2015_405_MOESM6_ESM.tif]
