# Supplementary material for: Morphological and ecological divergence of Lilium and Nomocharis within the Hengduan Mountains and Qinghai-Tibetan Plateau may result from habitat specialization and hybridization
Source: BMC Evol Biol. 2015 Jul 29;15:147. doi: 10.1186/s12862-015-0405-2 (PMC4518642; doi:10.1186/s12862-015-0405-2)
Supplement: Additional file 7: Figure S7. — Results of KH tests for ITS and combined plastid datasets. [file 12862_2015_405_MOESM7_ESM.pdf]

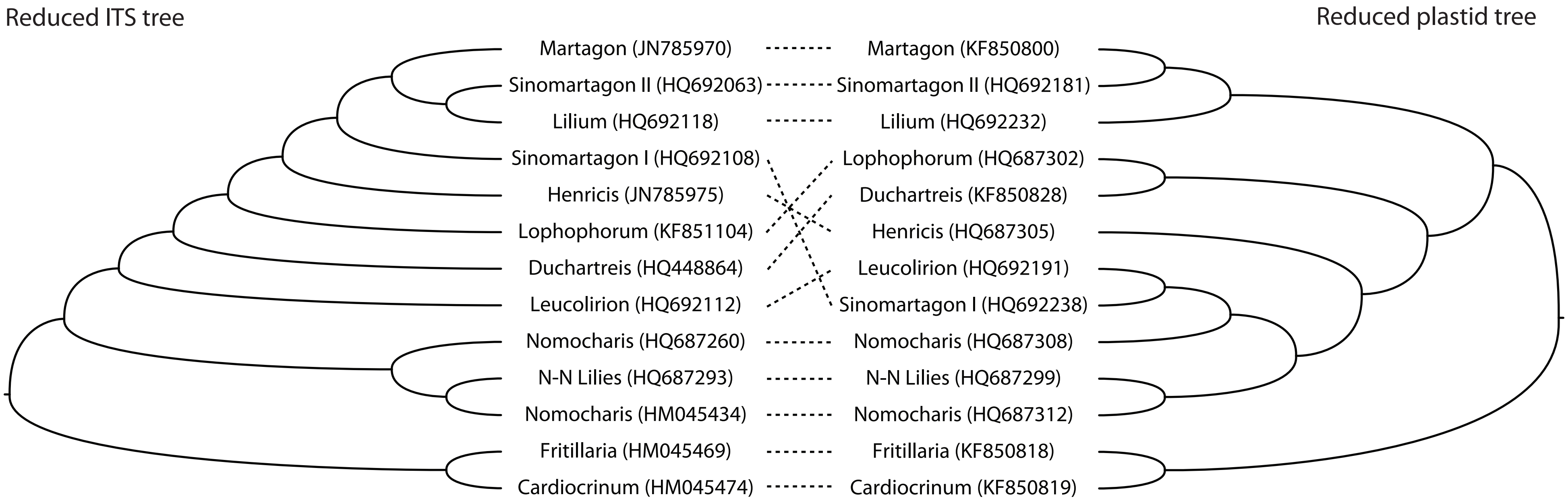

Reduced ITS and plastid cladograms showing the reconstructed relationships between the evolutionary lineages of *Lilium-Nomocharis.* Topologies based on the maximum clade credibilty trees. Dashed lines connect like lineages. Terminal names are comprised of lineage names and Genbank accession numbers for reference. In theplastid tree, the Genbank accession numbers are for *mat*K only. However, the tree represents the combined plastid topology.

	**ITS**		**plastid**	
	**-ln L**	**p-value**	**-ln L**	**p-value**
**Reconstructed Topology**	2057.57	<0.01	1553.95	<0.01
**Alternate Topology**	2109.40		1554.37	

Table showing the results of reciprocal comparisons between the ITS and plastid topologies shown above. Reconstructed topology refers to the reduced maximum clade credibility tree for the marker. Alternate topology refers to the reduced maximum clade credibility tree reconstructed using the other marker. One-tailed KH tests were used to compare likelihoods. In both cases, the reconstructed topology was significantly better than the alternatve. P-values are shown adjacent to favored topologies.
